# Improved resilience and proteostasis mediate longevity upon DAF-2 degradation in old age

**DOI:** 10.1007/s11357-024-01232-x

**Published:** 2024-06-20

**Authors:** Adrian Molière, Ji Young Cecilia Park, Anita Goyala, Elena M. Vayndorf, Bruce Zhang, Kuei Ching Hsiung, Yoonji Jung, Sujeong Kwon, Cyril Statzer, David Meyer, Richard Nguyen, Joseph Chadwick, Maximilian A. Thompson, Björn Schumacher, Seung-Jae V. Lee, Clara L. Essmann, Michael R. MacArthur, Matt Kaeberlein, Della David, David Gems, Collin Y. Ewald

**Affiliations:** 1 Laboratory of Extracellular Matrix Regeneration, Institute of Translational Medicine, Department of Health Sciences and Technology, ETH Zürich, CH-8603 Schwerzenbach, Switzerland; 2 Department of Laboratory Medicine and Pathology, University of Washington, Seattle, WA 98195-7470 USA; 3 Institute of Healthy Ageing, and Research Department of Genetics, Evolution and Environment, University College London, London, UK; 4 Department of Biological Sciences, Korea Advanced Institute of Science and Technology, 291 Daehak-ro, Yuseong-gu, Daejeon, 34141 South Korea; 5 Institute for Genome Stability in Aging and Disease, Medical Faculty, University Hospital and University of Cologne, Joseph-Stelzmann-Str. 26, 50931 Cologne, Germany; 6 Cologne Excellence Cluster for Cellular Stress Responses in Aging-Associated Diseases (CECAD), Center for Molecular Medicine Cologne (CMMC), University of Cologne, Joseph-Stelzmann-Str. 26, 50931 Cologne, Germany; 7 The Babraham Institute, Cambridge, UK; 8 Bioinformatics and Molecular Genetics, Institute of Biology III, Faculty of Biology, Albert-Ludwigs-University Freiburg, 79108 Freiburg, Germany; 9 Lewis-Sigler Institute for Integrative Genomics, Princeton University, Princeton, NJ 08540 USA

**Keywords:** End-of-life, Frailty, Insulin/IGF-1 signaling, Longevity, Protein homeostasis, Resilience

## Abstract

Little is known about the possibility of reversing age-related biological changes when they have already occurred. To explore this, we have characterized the effects of reducing insulin/IGF-1 signaling (IIS) during old age. Reduction of IIS throughout life slows age-related decline in diverse species, most strikingly in the nematode *Caenorhabditis elegans*. Here we show that even at advanced ages, auxin-induced degradation of DAF-2 in single tissues, including neurons and the intestine, is still able to markedly increase *C. elegans* lifespan. We describe how reversibility varies among senescent changes. While senescent pathologies that develop in mid-life were not reversed, there was a rejuvenation of the proteostasis network, manifesting as a restoration of the capacity to eliminate otherwise intractable protein aggregates that accumulate with age. Moreover, resistance to several stressors was restored. These results support several new conclusions. (1) Loss of resilience is not solely a consequence of pathologies that develop in earlier life. (2) Restoration of proteostasis and resilience by inhibiting IIS is a plausible cause of the increase in lifespan. And (3), most interestingly, some aspects of the age-related transition from resilience to frailty can be reversed to a certain extent. This raises the possibility that the effect of IIS and related pathways on resilience and frailty during aging in higher animals might possess some degree of reversibility.

**Supplementary information:**

The online version contains supplementary material available at 10.1007/s11357-024-01232-x.

## Introduction

Progressive functional decline is a defining characteristic of the aging process, manifesting across molecular, cellular, tissue, and organismal levels. As the organism ages, it becomes frailer, and the prevalence of various diseases, as well as overall mortality risk, rises sharply. The objective of geroscience, or aging research, is to understand the biological aging process and utilize this knowledge to promote health during old age [1]. This objective may be realized through the implementation of strategies that attenuate or prevent biological deterioration and also, more ambitiously, that reverse it. While much progress has been made in understanding the aging process and slowing biological decline in model organisms [2, 3], relatively little is known about the possibility of reversing age-related changes.

Across various model organisms, inhibition of the nutrient-sensitive and growth-promoting insulin/IGF-1 and mTOR signaling pathways results in the largest and most reproducible increases in healthspan and lifespan [4]. Key findings leading to these discoveries included the discovery that mutations in the genes *age-1* and *daf-2* dramatically extend *C. elegans* lifespan [5, 6]. These genes were later shown to encode components of the insulin/IGF-1 signaling (IIS) pathway; for example, the DAF-2 protein resembles the insulin and IGF-1 receptors [7]. Besides extending lifespan, reduced IIS also slows the age-related deterioration in biological function and health [8–12]. IIS regulates many biological processes, including embryonic and larval development, reproduction, motility, metabolism, inter-tissue signaling, immunity, stress defense, and protein homeostasis, including extracellular matrix remodeling [13–20].

In *C. elegans*, inhibition of IIS gene function was in the past usually achieved by means of mutation or RNA-mediated interference (RNAi). The efficacy of *daf-2* RNAi diminishes with age, which meant that the effects of late-life inhibition of IIS were not possible to test [21]. To address this problem, we previously utilized a system for the conditional auxin-mediated degradation (AID) of DAF-2 [22]; similar systems were developed by two other research groups [23, 24]. In these systems, expression of a modified *Arabidopsis* TIR1 F-box protein mediates auxin-induced degradation (AID) of degron-tagged targets, here DAF-2 [22, 25]. The AID system makes it possible to selectively reduce IIS at any point in life in any desired target tissue. Our previous study demonstrated that, surprisingly, reduced IIS robustly extends lifespan at any age, even in very old *C. elegans* in a state of advanced decrepitude [22].

**Fig. 1:**
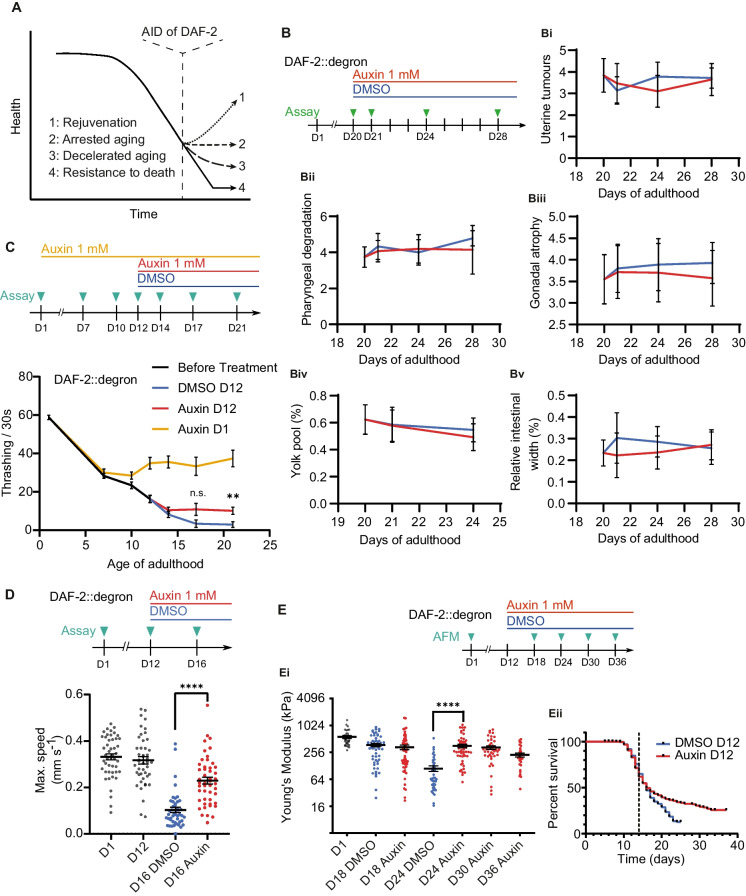
Late-life DAF-2 degradation partially extended classical healthspan markers without rejuvenation. **A** Scheme depicting the potential effects of late-life DAF-2 AID on *C. elegans* healthspan. 1: Rejuvenation, 2: Arrested aging, 3: Decelerated aging, 4: Resistance to death. **B** DAF-2 AID at day 20 of adulthood does not affect senescent pathologies. Whole-body DAF-2::degron *C. elegans* were treated with auxin or DMSO to a final concentration of 1 mM at day 20 and subsequently scored on the following days for uterine tumors (**Bi**), pharyngeal degradation (**Bii**), and gonadal atrophy (scored on a scale from 1 to 5, **Biii**), as well as the relative size of yolk pools (**Biv**) and intestinal width (**Bv**). **C** Decline in body thrashing rate arrested after DAF-2 AID at either day 1 or day 12 of adulthood compared to DMSO-treated control. Whole-body DAF-2::degron *C. elegans* were treated with auxin on day 1 or with either DMSO or auxin to a final concentration of 1 mM on day 12 and subsequently scored on days 14, 17, and 21. Error bars represent mean and SEM. Comparison of auxin and DMSO treatment from day 12: **p = 0.0042; n.s: *p* = 0.0717. Determined using Kruskal–Wallis test followed by Dunn’s multiple comparisons test. One out of three biological repeats is shown. For individual repeats and statistics, see Source Data File 1. **D** Decline in maximum movement speed slowed after DAF-2 AID on day 12 of adulthood compared to DMSO-treated control. Whole-body DAF-2::degron *C. elegans* were treated with either DMSO or auxin to a final concentration of 1 mM on day 12 and subsequently scored on day 16. Error bars represent mean and SEM. Comparison of auxin and DMSO treatment from day 12: ****p < 0.0001. Determined using a two-tailed unpaired *t*-test. One out of three biological repeats is shown. For individual repeats and statistics, see Source Data File 1. **E** DAF-2 AID on day 12 leads to the retention of youthful *C. elegans* stiffness based on atomic force microscopy. Shown are the Young’s modulus (**Ei**) and the lifespan (**Eii**) until *C. elegans* were assayed. Whole-body DAF-2::degron *C. elegans* were treated with either DMSO or auxin to a final concentration of 1 mM on day 12 and subsequently scored on days 18, 24, 30, and 36. Day 1 values represent untreated control. Error bars represent mean and SEM. ****p < 0.0001. Determined using a two-tailed unpaired *t*-test. Each dot represents Young’s modulus of individual *C. elegans*. Also shown are the survival curves of the same *C. elegans* used for probing the stiffness. The dotted black line indicates the onset of treatment. Pooled result of 3 independent biological trials. The increase in lifespan reached statistical significance in two out of three repeats. For additional information and individual repeats, see Source Data File 1 and Supplementary Table 1

We formulated four hypotheses to explain how *C. elegans* avoids death following late-life DAF-2 AID (Fig. 1A). The first hypothesis proposes that there is functional rejuvenation, leading to improved health after DAF-2 AID (i.e., the rejuvenation hypothesis (1)). The second suggests that aging is arrested, halting further deterioration of health but without rejuvenation (i.e., the arrested aging hypothesis (2)). The third suggests a continued but decelerated decline (i.e., the deceleration hypothesis (3)). The fourth hypothesis posits that there is continuous deterioration of health and physiological function but this fails to cause death (i.e., the death resistance hypothesis (4)).

In this study, we demonstrate the occurrence of decelerated decline without rejuvenation for functional age-related phenotypes following late-life DAF-2 AID. However, the capacity to survive certain stressors like heat and osmotic stress can get reactivated at old age to levels seen in young animals and beyond. Protein homeostasis (proteostasis) specifically showed marked reactivation potential, as evident by the clearance of otherwise irreversibly aggregated proteins after DAF-2 AID. The susceptibility of the *C. elegans* proteostasis-assurance mechanism to reactivation suggests the possibility that equivalent effects might be seen in higher animals. If that were the case, this could open the way to therapeutic interventions aimed at enhancing resilience and promoting health during aging in humans.

## Results

### Late-life DAF-2 AID increased lifespan without rejuvenation of health markers

In our previous work, we demonstrated that DAF-2 AID in geriatric *C. elegans* leads to a large increase in lifespan [22]. A major cause of death of *C. elegans* in the laboratory is undigested bacteria proliferating in the intestine or pharynx during old age [26, 27]. The common laboratory-used bacterial food source provided, *E. coli*, is mildly pathogenic to *C. elegans*, contributing to death in elderly animals [28, 29]. This raises the possibility that the increased lifespan after late-life DAF-2 AID could be due, at least in part, to resistance to death from bacterial infection. If this were the case, one would expect that DAF-2 AID would result in a small increase in lifespan in *C. elegans* maintained under conditions that prevent bacterial infection. To test this, we used two methods to run the lifespans on (1) dead bacteria and (2) antibiotics to inhibit bacterial proliferation. We found that DAF-2 AID at mid- or late-life was sufficient to increase the lifespan even when fed dead bacteria, suggesting a bacteria-resistance-independent lifespan extension (Supplementary Fig. 1A–B, Supplementary Table 1). Concomitantly, DAF-2 AID together with carbenicillin treatment at day 20 increased the lifespan of *C. elegans* markedly further than carbenicillin treatment alone. Similarly, late-life DAF-2 AID in the presence of carbenicillin from birth further extended the lifespan (Supplementary Fig. 1C, 1D, Supplementary Table 1). This suggests that resistance to bacterial proliferation is sufficient to mildly increase lifespan but plays a minor role and cannot explain the larger DAF-2 AID-mediated late-life lifespan extension. Thus, the question of which underlying mechanisms drive DAF-2 AID-mediated late-life lifespan extension remains unresolved.

*C. elegans* aging is morphologically characterized by the development of senescent pathologies that increase in severity with age and plateau between day 12 and day 15 of adulthood [30, 31]. To investigate the possibility of phenotypic rejuvenation following late-life DAF-2 AID, we monitored the development of 5 markers of senescent pathologies that have been previously described by Ezcurra and colleagues, which cover a broad spectrum of morphological aging-associated changes, namely uterine tumors, gonadal atrophy, deterioration of the pharynx (foregut), yolky lipoprotein pools in the body cavity (pseudocoelom), and the intestinal width [30]. After the manifestation of these senescent pathologies, we found no change in pathology levels upon mid-life (day 10 and 14) nor late-life (day 20) DAF-2 AID (Fig. 1B and Supplementary Fig. 2), suggesting that these alterations might be beyond repair. Furthermore, given that late-life DAF-2 AID extends lifespan, this observation also suggests that the reversal of these age-related pathologies is not necessary for late-life lifespan extension.

We next asked whether DAF-2 AID can lead to physiological rejuvenation using standard markers of *C. elegans* healthspan. As locomotion decreases with age, retention of higher movement rates is commonly assessed as a healthspan marker [10, 11, 32–34]. To assess locomotion capacity, we measured the rate of body thrashing in liquid following DAF-2 AID from day 12 of adulthood, a time when locomotion capacity is diminished but still present. This was found to modestly slow the subsequent age-related decline of locomotion capacity (Fig. 1C), consistent with the deceleration hypothesis. Similarly, we found that DAF-2 AID on day 12 slowed the decline in maximum movement speed (Fig. 1D), a predictive marker of lifespan [9]. By contrast, the rate of pharyngeal pumping, which also declines with age [34], was not slowed or restored by late-life DAF-2 AID (Supplementary Fig. 3). These data suggest that the age-related decline of some neuromuscular phenotypes was decelerated but not rejuvenated upon late-life DAF-2 AID.

Next, we assessed the biomechanical properties that change during aging. The abundance of collagens and other extracellular matrix components decreases with age, and in *daf-2* mutants, prolonged collagen enhancement promotes lifespan extension [13]. Moreover, cuticle integrity and stiffness of *C. elegans* gradually decline with age, such that changes in *C. elegans* stiffness provide a readout for health [35]. Notably, older *daf-2* mutants retain a more youthful stiffness relative to wild-type *C. elegans* of the same age [35]. We found that DAF-2 AID at day 12 of adulthood was sufficient to decelerate the decline in stiffness and to maintain youthful tissue mechanics, as well as retaining their cuticle integrity measured as roughness until day 30 of adulthood (Fig. 1E, Supplementary Fig. 4).

**Fig. 2:**
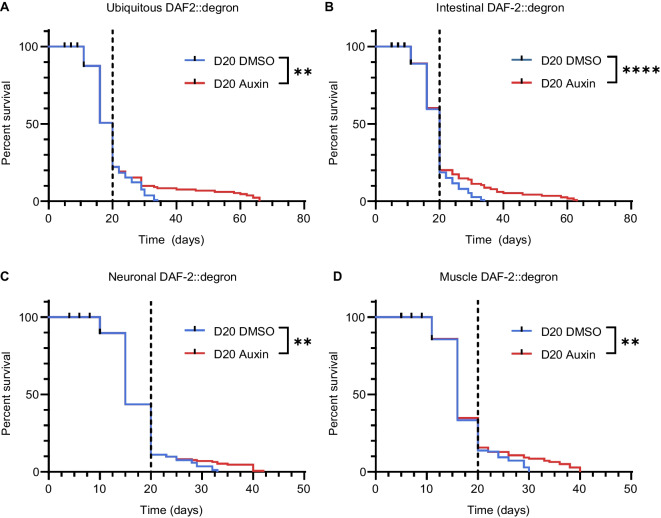
Late-life DAF-2 degradation in the intestine, neurons, and muscles was each sufficient for lifespan extension. Lifespans at 20 °C after DAF-2 AID at day 20 of adulthood in the whole body (ubiquitous, **p = 0.0036, **A**), intestine (****p < 0.0001, **B**), neurons (**p = 0.0024, **C**), and body wall muscle (**p = 0.0018, **D**). On day 20 of adulthood, DAF-2::degron *C. elegans* were top-coated with either DMSO (control) or auxin to a final concentration of 1 mM. Black dotted lines indicate the treatment timepoint. Statistics were determined using log-rank (Mantel-Cox) pairwise comparison. Two independent biological repeats were carried out. For additional information, see Supplementary Table 1

In summary, our findings confirm the lifespan extension observed with late-life DAF-2 AID (Supplementary Fig. 1, Fig. 1E, Fig. 2A). While there was no effect on markers of senescent pathologies, we found that markers of *C. elegans*’ locomotion and health showed an arrest or decreased pace in their further decline upon AID of DAF-2 (Fig. 1C–1E), with only pharyngeal pumping rate being unaffected (Supplementary Fig. 3) and thus providing evidence for the arrested and decelerated aging hypotheses (Fig. 1A).

### Late-life degradation of DAF-2 in targeted tissues was sufficient for lifespan extension

To shed light on the interconnected processes of the late-life DAF-2 AID-mediated lifespan extension (Fig. 2A), we investigated which tissues are necessary for this effect. Previous studies have revealed that AID-mediated degradation of DAF-2 in the intestine is sufficient to increase the lifespan of *C. elegans*, with a similar or smaller effect observed in neurons from early adulthood [22, 23]. We found that DAF-2 AID from day 20 of adulthood, restricted to muscle, neurons, or the intestine was each sufficient to increase lifespan (Fig. 2B–D). This was surprising but might be in part mediated by counteracting the gradual increase of DAF-2 protein levels during aging [36]. Therefore, in contrast to depleting IIS early in life where IIS might be still needed for growth, these results suggest that reducing IIS in geriatric ages in any tissue might be beneficial.

### Specific transcriptional changes implicated in lifespan increase upon late-life DAF-2 AID

**Fig. 3:**
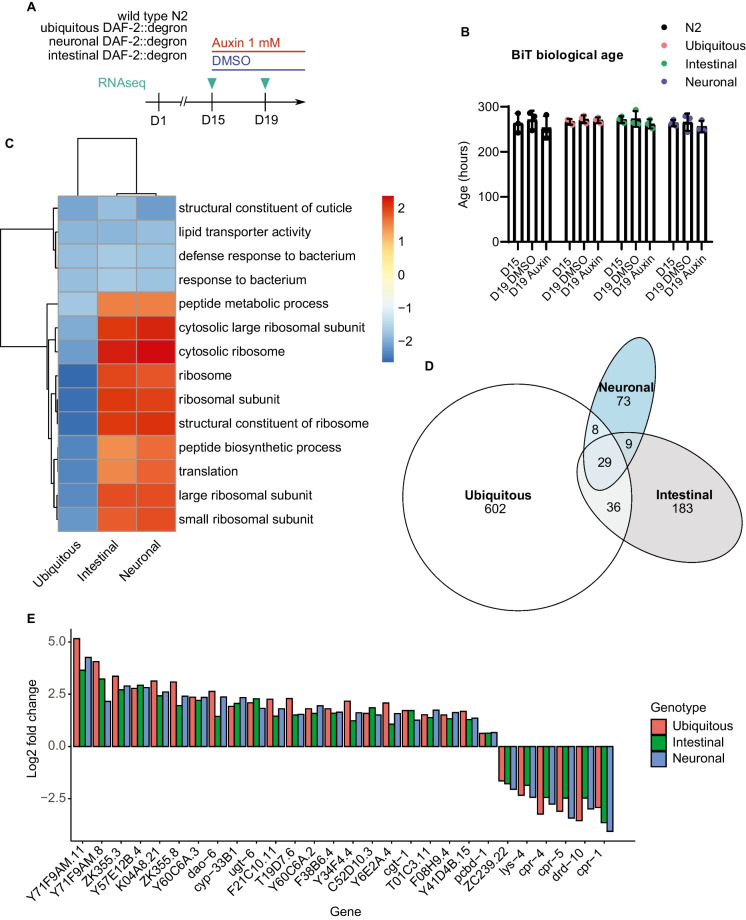
Specific changes in the transcription of stress resistance-related genes without global transcriptomic rejuvenation. **A** Scheme depicting the experimental procedure. Whole-body, intestinal, and neuron-specific DAF-2::degron as well as wild-type N2 *C. elegans* were used for RNAseq both before auxin/DMSO treatment on day 15 and after treatment on day 19. For additional information on the results, see Supplementary Discussion and Supplementary Table 2. **B** No changes in biological age based on the BiT age transcriptional clock in any of the *C. elegans* strains used after auxin (1 mM) treatment. Error bars represent mean and SEM. **C** Heatmap of differentially expressed genes grouped in GO terms, the scale represents fold-changes. **D** The number of significantly differentially expressed genes for each DAF-2::degron *C. elegans* strain and their overlap. **E** The differentially expressed genes shared between the three DAF-2::degron *C. elegans* strains (ubiquitous DAF-2::degron, neuronal DAF-2::degron, intestinal DAF-2::degron). See Supplementary Table 2 and Supplementary Discussion for details

Although it is interesting that late-life DAF-2 AID in muscles was also sufficient to increase lifespan, we decided to focus on the intestine and neurons, since in these tissues DAF-2 is strongly expressed [37] and reduced IIS in these tissues increases lifespan and stress resistance in early life, suggesting a positive action of reduced IIS is driving this rather than simply restoring dysregulated age-related IIS or nutrient-sensing. To identify potential shared underlying mechanisms that contribute to lifespan extension in old age upon neuronal, intestinal, and ubiquitous DAF-2 AID, we performed RNA sequencing (Fig. 3A). First, we asked whether the global transcriptome changes to a more youthful state after the late-life DAF-2 AID. For this, we used the neuronal, intestinal, and ubiquitous DAF-2::degron, as well as wild-type N2 *C. elegans,* and treated them with auxin or DMSO as a control on day 15 of adulthood. We harvested these *C. elegans* strains on day 15 (before treatment) and day 19 (post-treatment) of adulthood and performed RNA sequencing (Fig. 3A). Using two different aging clocks, the BiT age transcriptomic clock, or a stochastic data-based transcriptomic clock [38, 39], we found no difference in the transcriptional age in any of the strains after late-life DAF-2 AID (Fig. 3B; Supplementary Discussion for detailed explanations, Supplementary Table 2). However, during the period from day 15 to day 19, we also did not observe any signs of aging in the control groups. This observation may be attributed to the fact that younger ages (1–12 days of adulthood) were more highly represented in the training data used to generate the transcriptional clock and our lack of understanding of the transcriptional changes that occur at advanced ages. Additionally, the survivor bias of biologically younger *C. elegans* in the population is getting stronger with proceeding chronological age. Although BiT age tries to correct this, it might still underestimate the survivor bias, leading to an underestimation of the biological age in older populations (more details are discussed in the Supplementary Discussion).

Next, we analyzed the specific transcriptional changes seen after the DAF-2 AID. A gene ontology analysis revealed that the greatest transcriptional changes were seen in pathways related to protein translation, bacterial defense, and cuticle structure. Contrary to expectations, an opposite effect was observed in pathways related to protein translation between ubiquitous DAF-2::degron and the neuronal- and intestinal-specific strains (Fig. 3C). Furthermore, these alterations do not align with the effects of DAF-2 AID at day 1 of adulthood, based on data from Zhang et. al. [23] (Supplementary Fig. 5A), with an inverse effect on cuticular and immunity-related genes. This suggests that the age of onset strongly influences the transcriptomic signature. The relative magnitude of transcriptional changes after DAF-2 AID on day 15 among the strains differed, with ubiquitous DAF-2 AID showing the greatest change in the number of differentially expressed genes (Fig. 3D). This is consistent with the changes seen upon DAF-2 AID on day 1 (Supplementary Fig. 5B). Only a relatively small number of genes (29 genes) were differentially expressed with the same directionality between the whole-body and tissue-specific DAF-2::degron strains (Fig. 3D). Since DAF-2 degradation in the intestine and neurons was sufficient to extend lifespan, we further investigated the overlapping genes between those three strains (Fig. 3E). The majority of the genes were uncharacterized; however, of the characterized ones, we found genes related to stress resistance, in particular proteostasis and heat stress, oxidative and radiation stress, and bacterial defense (Specifics in Supplementary Discussion). Interestingly, even though the transcriptomic signature differs between DAF-2 AID in young (day 1) and old (day 15) animals, there is considerable similarity between the specific genes that are consistently changed across the three tissue models and different ages (Supplementary Fig. 5C–F).

In summary, we find relatively little transcriptomic changes in response to DAF-2 AID at day 15 of adulthood, thus implying that a major remodeling of the transcriptome might not underlie the late-life lifespan extension upon DAF-2 AID.

### DAF-2 AID can reactivate the capacity to survive stress

**Fig. 4:**
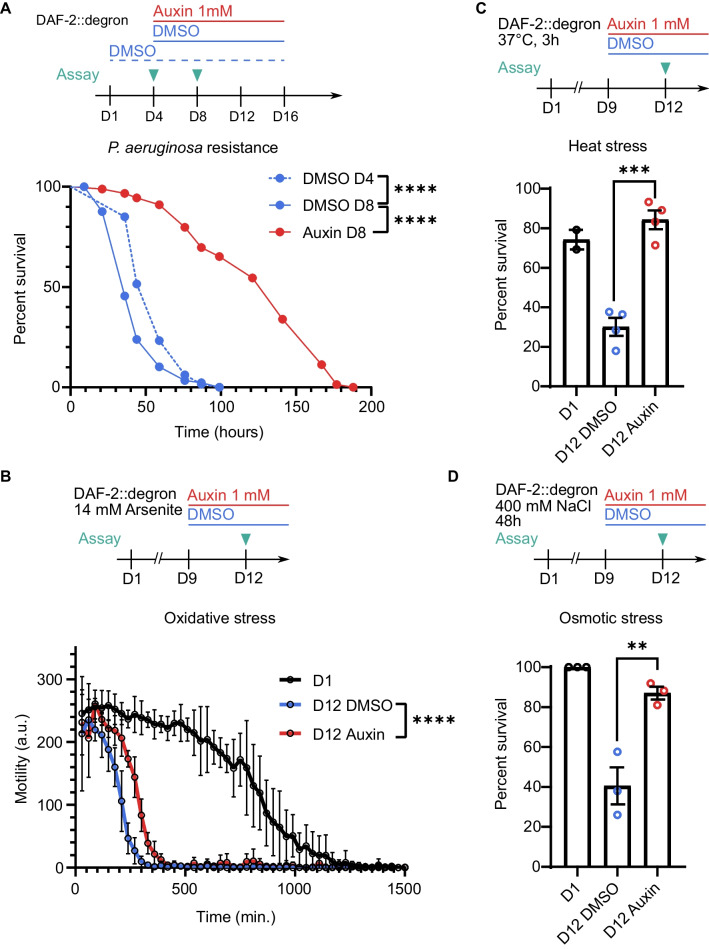
Reactivation of stress resistance capacity upon DAF-2 AID. **A** DAF-2 AID on day 8 of adulthood boosted whole-body DAF-2::degron *C. elegans* infection survival under *Pseudomonas aeruginosa* exposure compared to DMSO-treated on day 4 and day 8 of adulthood. *C. elegans* were transferred to DMSO or 1 mM auxin-containing plates coated with *Pseudomonas aeruginosa* on either day 4 or day 8 as indicated and subsequently scored for survival. ****p < 0.0001 was determined using log-rank (Mantel-Cox) pairwise comparison. For additional information, see Source Data File 1. **B** DAF-2 AID on day 12 of adulthood partially rescued age-related loss of oxidative stress resistance in *C. elegans* compared to DMSO-treated controls. Whole-body DAF-2::degron *C. elegans* were transferred to either DMSO or 1 mM auxin-containing plates on day 9. On day 12, animals were transferred to 14 mM arsenite-containing wells, and locomotion capacity was measured automatically every hour using the wMicroTracker system. Error bars represent mean and SD. For statistics, the area under the curve was compared with an unpaired, two-tailed *t-*test. For additional information, see Source Data File 1. **C** DAF-2 AID on day 12 reversed the age-related loss of heat stress resistance in *C. elegans* compared to DMSO-treated controls on day-12 or day-1-old adult animals. Whole-body DAF-2::degron *C. elegans* were transferred to either DMSO or 1 mM auxin-containing plates on day 9. Three days later, on day 12, *C. elegans* were transferred to new DMSO or 1 mM auxin-containing plates, heat-shocked for 3 h at 37 °C, and scored for survival 24 h later. Error bars represent mean and SEM. ***p < 0.001. Each dot represents a pooled survival rate for one biological repeat. For additional information, see Source Data File 1. **D** *C. elegans* DAF-2 AID on day 12 reversed the age-related loss of osmotic stress resistance in *C. elegans* compared to DMSO-treated controls on day 12 or day 1 old adult animals. Whole-body DAF-2::degron *C. elegans* were transferred to either DMSO or 1 mM auxin-containing plates on day 9. On day 12, DAF-2::degron *C. elegans* were transferred to fresh 400 mM NaCl plates that also contained DMSO or 1 mM auxin, and scored for survival 48 h later. Error bars represent mean and SEM. **p < 0.01. Each dot represents a pooled survival rate for one biological repeat. For additional information, see Source Data File 1

Next, we asked whether the observed specific transcriptional changes upon late-life DAF-2 AID correlate with respective functional improvement, in particular the ability to survive stress. For this, we degraded DAF-2 at ages when the resistance of *C. elegans* to a certain stressor has shown age-related decline and then tested whether the capacity to survive those stressors can be restored. Our transcriptomic analysis highlighted changes in the defense against bacterial infection (Fig. 3C, E, Supplementary Discussion). The immune response to various pathological bacterial infections decreases with age in *C. elegans*, with 7-day-old adults already showing more rapid death in response to several pathogenic bacteria [40]. DAF-2 mutants show increased pathogen resistance and substantially delayed immunosenescence, and the downstream transcription factor DAF-16 induces several antimicrobial genes [18, 20, 41]. We found that on day 8 of adulthood, resistance to pathogenic *Pseudomonas aeruginosa* was decreased in control conditions and that DAF-2 AID greatly improved bacterial defense, well beyond day 4 levels (Fig. 4A). Thus, degrading DAF-2 counteracts post-reproductive immunosenescence.

To investigate whether, after their age-related decline, resistance to other stressors can also get reactivated, we asked whether DAF-2 AID can recover the ability to survive oxidative stress, which decreases with age [42, 43]. We observed that DAF-2 AID applied on day 9 of adulthood and assayed three days later increased the resistance to oxidative-damage-inducing arsenite, although not to the level seen in young animals (Fig. 4B).

The capacity to survive osmotic stress and mild heat stress gradually declines with age in *C. elegans* [42, 44]*.* Following DAF-2 AID started on day 9 of adulthood, the capacity to recover from mild heat stress (3 h, 37 °C) and osmotic stress (400 mM NaCl) was markedly improved three days later (on day 12), comparable with the survival of young animals (Fig. 4C, D). Next, we asked whether the lifespan extension upon AID of DAF-2 at very old age (day 20 of adulthood) is associated with restoration of stress resistance. Remarkably, there was still a strong reversal of stress resistance at day 24 of adulthood following AID of DAF-2 at day 20 of adulthood (Supplementary Fig. 6). This was the first experimental evidence supporting the proposed rejuvenation hypothesis (Fig. 1A).

Resistance to both heat and osmotic stress and to a lesser extent oxidative stress requires functional protein homeostasis (proteostasis) mechanisms [45–47]. Proteostasis is maintained by a complex network that controls protein synthesis, folding, and degradation. However, these systems become less efficient with age [45]. Heat stress leads to acute protein damage and aggregation, which becomes lethal with impaired proteostasis in old age. Although the specific osmotic stress response mechanisms are unique, hyperosmotic stress also leads to a major disruption of proteostasis and rapid protein aggregation [46, 48, 49]. Thus we hypothesized that DAF-2 AID in old age leads to a reactivation of parts of the proteostasis network, thereby clearing otherwise lethal protein damage and improving the ability to recover from stress.

### Degradation of DAF-2 reversed the age-related aggregation of endogenous proteins

To investigate the possible reactivation of the proteostasis network, we asked whether DAF-2 AID can reverse the age-related aggregation of proteins. An often-used model for disease-related protein aggregation is exogenous polyglutamine with 35 repeats fused to YFP (polyQ35::YFP) [50]. Following DAF-2 AID on day 8 of adulthood, at which timepoint the *C. elegans* showed high levels of polyQ35::YFP aggregation, we observed a reduction in overall intensity but not in total aggregate numbers, even though DAF-2 degradation was sufficient to increase the lifespan of these transgenic animals (Supplementary Fig. 7). This is consistent with previous studies showing an increased human disease-associated protein aggregation in the body wall muscles of *daf-2* deficient *C. elegans* [51–53], and indicates that removal of these artificial human disease-associated YFP-reporter protein aggregates is not necessary for lifespan extension following DAF-2 AID.

**Fig. 5:**
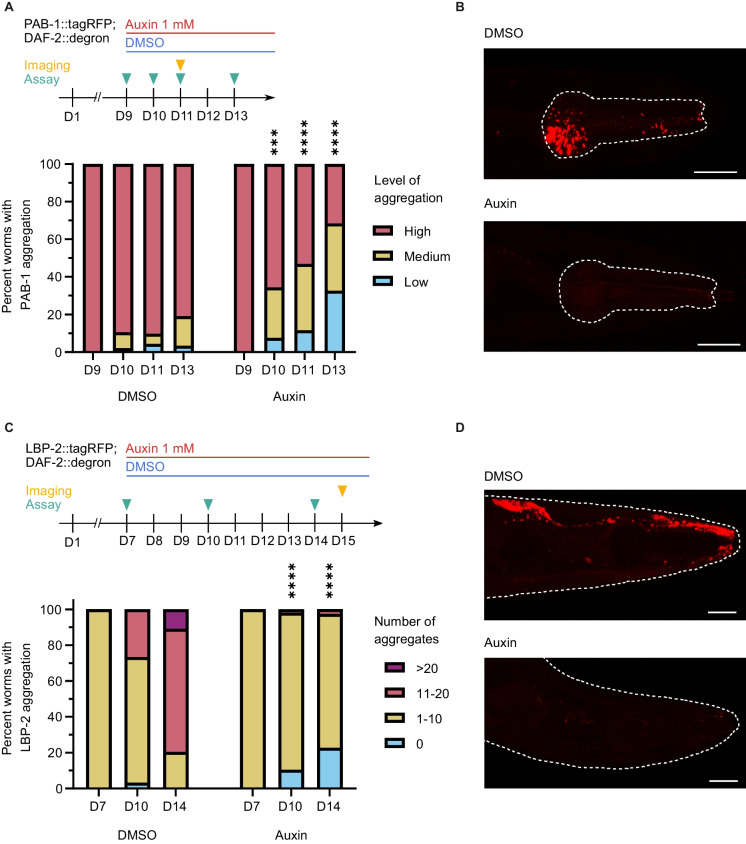
Clearance of aggregated endogenous proteins following DAF-2 AID. **A** DAF-2 AID on day 9 in whole-body DAF-2::degron; PAB-1::tagRFP *C. elegans* with high PAB-1 aggregation leads to reversal of PAB-1 aggregation compared to DMSO-treated control. On day 9, *C. elegans* with high levels of PAB-1 aggregation were selected and split into two groups, which were transferred to either DMSO or 1 mM auxin-containing plates. The PAB-1 aggregation status of each *C. elegans* was subsequently scored on days 10, 11, and 13. ****p < 0.0001; ***p < 0.001. Determined using the Chi-Square test. For additional information, see Source Data File 1. **B** Representative images of whole-body DAF-2::degron; PAB-1::tagRFP *C. elegans* on day 11 that had a high level of PAB-1 aggregation at D9 prior to DAF-2 AID or DMSO treatment. Procorpus and anterior pharyngeal bulb are outlined. Scale bar = 20 µm. **C** DAF-2 AID on day 7 in whole-body DAF-2::degron; LBP-2::tagRFP animals with 1–10 LBP-2 aggregates, leads to an arrest and partial reversal of LBP-2 aggregation compared to DMSO-treated control. On day 7, *C. elegans* with 1–10 LBP-2 aggregates were selected and split into two groups, which were transferred to either DMSO or 1 mM auxin-containing plates. The LBP-2 aggregation status of each *C. elegans* was subsequently scored on days 8, 9, 10, 11, and 14. ****p < 0.0001. Determined using the Chi-Square test. For additional information, see Source Data File 1. **D** Representative images of whole-body DAF-2::degron; LBP-2::tagRFP *C. elegans* heads (outlined) at day 15 that had a medium level of LBP-2 aggregation (1–10 aggregates) at D7 prior to DAF-2 AID or DMSO treatment. Scale bar = 20 µm

Widespread endogenous protein aggregation occurs during normal aging, in the absence of additional stressors [54, 55]. We next looked at two endogenous proteins that naturally aggregate with age in *C. elegans*. PAB-1 is an RNA-binding protein with low-complexity prion-like domains that form intracellular solid aggregates with age in *C. elegans* [56]*.* It has been shown previously that reduced DAF-2 signaling slows down the progression of PAB-1 aggregation [56], but to the best of our knowledge, it has not been possible before to reverse already-formed PAB-1 aggregates. We found that on day 9 of adulthood, at a timepoint at which *C. elegans* showed high levels of fluorescent-tagged PAB-1::tagRFP aggregates in the pharyngeal muscles, DAF-2 AID effectively cleared these aggregates within four days (Fig. 5A). Excitingly, a subset of animals exhibited a complete absence of aggregates after the DAF-2 AID (Fig. 5A, B). We also observed clearance and reduced aggregation in the pharyngeal muscles with age of the faster aggregating protein RHO-1 by DAF-2 AID (Supplementary Fig. 8).

Next, we investigated if reduced *daf-2* signaling promotes restoration of extracellular proteostasis. For this, we used a model of age-dependent extracellular protein aggregation, where fluorescent-tagged LBP-2 aggregates extracellularly, being secreted by body wall muscles and forming aggregates in the pseudocoelom with age [57]. Our findings revealed that DAF-2 AID in aged animals exhibiting a moderate level of LBP-2::tagRFP aggregation in the head region (day 7 of adulthood) effectively arrested the formation of new aggregates (Fig. 5C). Similar to PAB-1::tagRFP, in a subset of animals, DAF-2 AID completely cleared them of aggregates, although the proportion of animals exhibiting complete clearance was smaller compared to the clearance observed with PAB-1::tagRFP aggregates (Fig. 5C, D).

In summary, as further supporting experimental evidence for the proposed rejuvenation hypothesis (Fig. 1A), following the degradation of DAF-2, we observed a reduction in the age-related aggregation of both intra- and extracellular endogenous proteins, suggesting a re-activation of the proteostasis network, which is consistent with the improvement of mild heat and osmotic stress resistance. Thus, the reactivation of the proteostasis network is likely to play a major role in the longevity resulting from late-life degradation of DAF-2.

## Discussion

Timing of longevity interventions is critical, however, only relatively little is known about interventions and potential rejuvenation in entire organisms during old age. Altered insulin/IGF-1 signaling (IIS) is among the strongest aging interventions in *C. elegans*, making it an ideal model to investigate to what extent rejuvenation might be possible during geriatric ages. Previously, we have shown that even at very old age, degradation of DAF-2 is sufficient to markedly increase *C. elegans* lifespan [22]. Here, we asked in which way the health and aging of *C. elegans* are affected by late-life DAF-2 AID. We demonstrated that late-life degradation of DAF-2 is not accompanied by a rejuvenation in overall health, based on classical health markers, or the transcriptome age clock of *C. elegans*. Rather, we showed that the decline of most physiological parameters was slowed and the disintegration of morphological structures was halted. Since with these markers we observed no rejuvenation but rather a slowing in decline, it is unlikely that they represent underlying mechanisms facilitating late-life DAF-2 AID-mediated longevity. However, we did observe a reactivation of proteostasis as evidenced by the clearance of age-related and endogenous protein aggregates alongside a marked improvement in protein stress resilience comparable to young animals even upon late-life DAF-2 AID on day 20. This suggests that the activation of proteostasis mechanisms plays a key role in late-life DAF-2 AID-mediated longevity.

It is well established that reduced insulin/IGF-1 signaling by congenital genetic mutations retards the decline in classical health markers, such as the pharyngeal pumping rate and locomotion capacity [5, 15]. However, it is not clear to what extent a decline can be reversed. We found that degrading DAF-2 in aged *C. elegans* with impaired locomotion capacity in the form of reduced bodily thrashing and maximum movement speed does not rejuvenate their locomotion, although the further decline is halted, which is in line with the hypothesis of an arrest in physiological decline upon degradation of DAF-2 (Fig. 1A, C, D). Consistent with this, the deterioration of stiffness and cuticle integrity, and hence the overall structural integrity is halted but not reversed upon the DAF-2 AID from day 12 of adulthood (Fig. 1E, Supplementary Fig. 4). It should be noted that the effect on healthspan markers could depend on the specific timing of the AID of DAF-2, with rejuvenation of these markers potentially possible at earlier timepoints.

Concomitantly, we observed relatively small changes in the global transcriptome. This suggests that the increased longevity upon late-life DAF-2 AID is largely mediated by post-transcriptional changes, which is in line with previous findings showing that stress resistance in *daf-2* mutants is mediated by post-transcriptional mechanisms [58]. In accordance, no change in the transcriptomic age based on the BiT age aging clock was observed upon late-life DAF-2 AID (Fig. 3B). However, due to the limitations described above and in the Supplementary Discussion, no apparent change in transcriptomic age could also be attributed to the lack of training data on old animals. It is unclear whether novel aging clocks that capture transcriptomic changes in old age would detect a change in transcriptomic age. However, it can be concluded that the transcriptome is not rejuvenated to the state of a young animal, on which the BiT age clock was trained [38]. This suggests that at least for lifespan extension through DAF-2 AID at late life a reversing to a youthful transcriptome is likely to play only a minor role. However, it cannot be excluded that AID of DAF-2 at earlier timepoints shows more pronounced changes in the transcriptome.

We demonstrated that upon DAF-2 AID, the age-related decline in survival to heat and osmotic stress can get boosted to levels seen in young animals, even when initiated at very old age (day 20 of adulthood), while the resistance to oxidative stress in the form of arsenite showed only modest improvement (Fig. 4B–D, Supplementary Fig. 6). Severe oxidative stress disrupts many aspects of normal cell function, affecting membranes, lipids, proteins, and DNA [59]. This suggests that the age-related decline in some, but not all, underlying mechanisms protecting against oxidative stress can be reactivated by the degradation of DAF-2, while the respective mechanisms dealing with heat- and osmotic stress seem more amendable at old age.

Hypertonic osmotic stress leads to an intracellular loss of water, cell shrinkage, ionic imbalance, and subsequent rapid protein damage and aggregation [48]. The immediate line of defense consists of the activation of the conserved ‘With No Lysine’ protein kinase 1 (WNK-1) to restore cell volume and upregulation of glycerol-3-phosphate dehydrogenase (GPDH-1), which leads to the synthesis of the protective osmolyte glycerol [60]. Secondly, *C. elegans* needs to deal with the protein damage and aggregation resulting from the osmotic stress [46]. Reduced DAF-2 signaling requires DAF-16 target genes involved in protein quality control to protect against lethal osmotic stress [61]. Insulin/IGF-1 signaling in itself is not osmotically regulated, nor required for adaptation to hyperosmotic conditions [61]. Thus, a broad activation of the proteostasis system is likely to protect *C. elegans* from otherwise lethal protein damage after the osmotic shock with prior degradation of DAF-2, explaining the markedly improved survival.

The effect of heat shock is characterized by a broad and direct disruption of proteostasis, including disrupted protein folding and function as well as aggregation [62]. This disruption is counteracted by reduced insulin/IGF-1 signaling, a major pathway in the heat shock response, involving both DAF-16 and heat-shock factor-1 (HSF-1) [48]. Notably, the significant overlap between genes promoting heat stress tolerance and modulating lifespan in *C. elegans* underscores the crucial role of proteostasis in aging and longevity [63, 64]. Furthermore, the restoration of survival capacity observed after both heat- and osmotic-induced shocks suggests a high degree of reversibility in the age-related collapse of proteostasis.

In *C. elegans,* the proteostasis network collapses at an early stage in adulthood, which is prevented by the modulation of DAF-16 [65–67]. The proteostasis network also declines with age in humans and plays a crucial role in age-related pathologies, with protein aggregation being a hallmark of neuropathologies like Parkinson’s and Alzheimer’s disease [68, 69]. As a consequence of the proteostasis disruption with age, part of the proteome becomes vulnerable to misfolded and intrinsically aggregation-prone proteins that accumulate in solid aggregates during normal aging, albeit during a longer timeframe compared to acute proteostressors, such as heat stress and osmotic stress [46, 51, 54, 55, 70–77]. Prior evidence shows that age-dependent protein aggregation is toxic and contributes to functional decline [78]. Moreover, reducing *daf-2* signaling during adulthood is a highly effective intervention to prevent age-dependent protein aggregation [56]. Our present findings reveal that late-life DAF-2 AID enables the organism to clear already-formed age-dependent solid aggregates and to survive stress related to protein damage (Fig. 5A–D). By eliminating protein aggregates and ultimately restoring intracellular and extracellular proteostasis, it is likely that protein quality control factors become more available to protect the proteome during periods of stress and aging, likely playing a role in the improved longevity following late-life DAF-2 AID. This is in line with previous findings showing that among 21 lifespan-extending drugs, only Minocycline retained its lifespan-extending effect when treated on day 8 of adulthood instead of day 1 [79]. This effect is attributed to the inhibition of protein aggregation without the activation of stress signaling pathways, highlighting the importance of proteostasis in late-life lifespan extension [79]. Similarly, genes involved in proteostasis were identified to extend lifespan when initiated post-reproductively on day 9 of adulthood in an RNAi screen [80]. Further investigation into the mechanisms underlying proteostasis network rejuvenation may offer novel therapeutic strategies for combating age-related disorders and improving overall health in older individuals.

In summary, at the beginning of this study, we postulated four hypotheses potentially explaining the effect of late-life DAF-2 AID on health, namely rejuvenation (1), arrested aging (2), decelerated aging (3), and resistance to death (4) (Fig. 1A). Our data suggests that most markers of health show a decelerated decline even at old age, however, without reversal or rejuvenation. On the other hand, we find a continued capacity for the activation of proteostasis mechanisms and adaptive stress response in old age. We propose that these mechanisms are likely to promote improved survival following the degradation of DAF-2 in geriatric *C. elegans.*

## Materials and methods

### *C. elegans* strains

*Caenorhabditis elegans* strains were maintained on NGM plates and OP50 Escherichia coli bacteria. The wild-type strain was N2 Bristol.

RAF2181 ubiquitous DAF-2::degron: *ieSi57* [*eft-3p*::TIR1::mRuby::*unc-54* 3′UTR + *Cbr-unc-119*( +)] II; *daf-2*(*bch-40*[degron-3xFLAG-STOP-SL2-SV40-degron-wrmScarlet-egl-13NLS]) *unc-119(ed3)* III.

RAF5033 Intestine-specific AID DAF-2::degron (alias RV6): *bchSi60*[P*vha-6*::TIR1::mRuby::*tbb-2*] II; *daf-2*(*bch-40*[degron-3xFLAG-STOP-SL2-SV40-degron-wrmScarlet-egl-13NLS]) *unc-119(ed3)* III.

RAF5032 Muscle-specific AID DAF-2::degron (alias RV7):

*bchSi59*[P*myo-3:*:TIR1::mRuby::*tbb-2*] II; *daf-2*(*bch-40*[degron-3xFLAG-STOP-SL2-SV40-degron-wrmScarlet-egl-13NLS]) *unc-119(ed3)* III.

RAF5034 Pan neuron-specific AID DAF-2::degron (RV8):

*bchSi64*[P*rab-3*::TIR1::mRuby::*tbb-2*] II; *daf-2*(*bch-40*[degron-3xFLAG-STOP-SL2-SV40-degron-wrmScarlet-egl-13NLS]) *unc-119(ed3)* III.

ARM415 ubiquitous DAF-2::degron with muscle-specific PolyQ35: *ieSi57* [*eft-3p*::TIR1::mRuby::*unc-54* 3′UTR + Cbr-unc-119( +)] II; *daf-2*(*bch-40*[degron-3xFLAG-STOP-SL2-SV40-degron-wrmScarlet-egl-13NLS]) *unc-119(ed3)* III; *rmIs132* [*unc-54p*::Q35::YFP].

DCD421: *ieSi57* [*eft-3p::TIR1::mRuby::unc-54* 3′UTR + *Cbr-unc-119(* +*)*] II; *daf-2(bch-40[degron-3xFLAG-STOP-SL2-SV40-degron-wrmScarlet-egl-13NLS]*) *unc-119(ed3)* III; N2B; *uqIs5[plbp-2::lbp-2::tagrfp*].

DCD423: *ieSi57* [*eft-3*p*::TIR1::mRuby::unc-54* 3′UTR + *Cbr-unc-119(* +*)*] II*; daf-2*(*bch-40* [*degron-3xFLAG-STOP-SL2-SV40-degron-wrmScarlet-egl-13NLS*]) *unc-119(ed3)* III; N2B; *uqIs24[Pmyo-2::tagrfp::pab1]*.

DCD434: *ieSi57* [*eft-3p*::TIR1::mRuby::*unc-54* 3′UTR + Cbr-unc-119( +)] II; *daf-2*(*bch-40*[degron-3xFLAG-STOP-SL2-SV40-degron-wrmScarlet-egl-13NLS]) *unc-119(ed3)* III; N2B; *uqIs12*[*pmyo2::rho-1::venus*].

### General *C. elegans* handling

All strains were maintained on NGM plates and OP50 *Escherichia coli* at 20 °C. For experiments, *C. elegans* were treated with FUdR (5-fluorodeoxyuridine, Sigma-Aldrich, F0503-1G) at a final concentration of 50 μM, unless specified otherwise.

### Auxin plates

Auxin plates were prepared as previously described [22]. Auxin (indole-3-acetic acid, Sigma #I3750) was dissolved in DMSO to prepare a 400 mM stock solution and stored at 4 °C. Auxin was added to NGM agar that had cooled down to about 60 °C before pouring the plates to a final concentration of 1 mM [25]. Control plates contained the same amount of DMSO (0.25% for 1 mM auxin plates).

### Lifespans

For late-life AID, auxin plates were prepared by topically adding 25 μL of auxin (400 mM) to 60 mm NGM plates, giving a final concentration of 1 mM. Control plates contained the same amount of DMSO, as previously performed [22]. For tissue-specific degradation of DAF-2, strains were used that selectively expressed TIR1 in muscles, neurons, or intestine, by using as drivers of expression *myo-3, rab-3*, and *vha-6* promoters, respectively. For lifespan assays, animals were maintained at 20 °C on 60 mm Petri dishes containing NGM agar seeded with 70 μl *E. coli* suspension. No FUdR was used, for the first 4 days of adulthood *C. elegans* were transferred daily onto fresh plates, and from day 5 *C. elegans* were every 2 days until reproduction had ceased. To score mortality, immobile animals were gently prodded with a platinum wire. Deaths due to animals crawling up the walls of the Petri dish and becoming desiccated, or to internal hatching of progeny, were treated as censored values in subsequent analysis.

For the lifespans of *C. elegans* fed dead bacteria, automated survival analysis was conducted using the lifespan machine setup described by [81]. For *C. elegans* preparation and details, see Statzer et al. (2022) [10]. Briefly, approximately 1000 L4 animals were resuspended in M9 and transferred to NGM plates containing 50 µM 5-Fluoro-2′deoxyuridine (FUdR) seeded with heat-killed OP50 bacteria (ca. 100–200 *C. elegans* per plate) and incubated at 20 °C until day 5 and 15 of adulthood. Animals were then resuspended (ca. 35–45 *C. elegans* per plate) in M9 and transferred to fresh FUdR plates containing either DMSO or 1 mM auxin tight-fitting lids (BD Falcon Petri Dishes, 50 3 9 mm), and the plates were dried with their lids open for 30 min after transfer. Air-cooled Epson V800 scanners were utilized for all experiments operating at a scanning frequency of one scan of 30 min. Temperature probes (Thermoworks, Utah, USA) were used to monitor the temperature on the scanner flatbed and kept constant at 20 °C.

For lifespans in the presence of carbenicillin, synchronized RAF2181 *C. elegans* were obtained by egg-lay, and lifespan experiments were performed without FUdR. Carbenicillin cohorts were transferred at the L4 stage to 600 mm (10 mL NGM) plates pre-treated one day prior with carbenicillin (80 μL, 500 mM) added around the lawn. Auxin-treated cohorts were transferred on day 20 of adulthood (D20) to 600 mm (10 mL NGM) plates pre-treated the day before (in addition to carbenicillin) with either auxin (25 μL, 400 mM in DMSO) or DMSO only (25 μL, 100%), added around the lawn. Survival was assessed by gently prodding *C. elegans* with platinum wire every 2–3 days; animals that did not move in response were scored as dead. The statistical significance of differences in mean lifespan was assessed by log-rank tests in JMP and graphs produced in GraphPad Prism (8.0.1).

### Body thrashing and pharyngeal pumping

To measure the pharyngeal pumping, the pumping rate of randomly selected *C. elegans* from a continuous population was manually counted over a time of 30 s. For the thrashing assay, individual *C. elegans* were transferred into a drop of physiological M9 buffer on a microscopy slide. After a period of acclimatization of 60 s, the number of full-body bends was counted for 30 s. If there was considerable variation in the pumping or thrashing rate over the 30 s (e.g., prolonged pause), then the measurement was repeated. Dead *C. elegans* were not assayed. Auxin or DMSO was top-coated at the indicated timepoints. Three biological repeats were performed for the thrashing assay and 2 for the pharyngeal pumping assay. For statistics, a Kruskal-Wallis test and Dunn’s multiple comparisons were performed in GraphPad Prism (8.0.1).

### Measurement of maximum movement speed

Maximum movement speed was adapted from Hahm et al. [9]. Age-synchronized adult DAF-2::degron (RAF2181) nematodes were synchronized by allowing egg laying on NGM plates for 3 h, followed by incubation of the eggs at 20 °C after removing the adults. Assay plates, prepared as NGM plates without a bacterial lawn, were used for velocity assays at 20 °C without lids. For each trial, over 200 *C. elegans* were grown, and on the day of the assay, 50 were transferred to individual assay plates for velocity measurements. After the day 12 measurement, *C. elegans* were divided into two groups for treatment with either DMSO (control) or auxin (1 mM final concentration) and measured again on day 16. For the measurement, after a short acclimation period, individual *C. elegans* movement was recorded for 30 s at 30 fps using a stereomicroscope (SMT1-FL-QC) with a USB 3.0 camera (DFK 23UX236) and the IC Capture 2.4 software, at a total magnification of × 15. Movement analysis was performed using ImageJ v1.53 k with the wrMTrck plugin, which calculates maximum speed. Data was subsequently analyzed in R (version 4.3.2) and plotted using GraphPad Prism (8.0.1).

### Measurement of senescent pathologies

RAF2181 *C. elegans* were maintained without FUdR and transferred to 600 mm (10 mL NGM) plates pre-treated the day before with either auxin (25 μL, 400 mM in DMSO) or DMSO only (25 μL, 100%). To image the senescent pathologies, 5–10 live nematodes per timepoint per condition were mounted onto 2% agar pads, anesthetized with a drop of 0.2% levamisole, and Nomarski images were captured at 630 × magnification with an ApoTome.2 Zeiss microscope with a Hamamatsu digital camera (C13440 ORCA-Flash4.0 V3) and the ZEN software. Brightness and contrast were adjusted equally across the entire image and, where applicable, applied equally to controls.

Pharynx pathology, uterine tumors, and gonad atrophy were classified into classes 1 to 5 according to the severity of their pathology: 1 = no visible pathology; 2 = subtle signs of deterioration; 3 = clearly discernible, mild pathology; 4 = well-developed pathology; and 5 = tissue so deteriorated as to be barely recognizable, such as the unrecognizable pharynx, large tumor that occupies the entire body width, or gonad that can no longer be identified. Intestinal atrophy and yolk pool scores were obtained using FIJI, using the following formulas, respectively: [(intestine width − lumen width)/*C. elegans* body width] × 100, and, (yolk pool area/body area) × 100. The statistical significance of differences between mean pathology scores was assessed by two-way ANOVA, and graphs were generated in GraphPad Prism (8.0.1).

### *C. elegans* total RNA sample preparation for transcriptomics

*C. elegans* strains wild-type (N2), ubiquitous DAF-2::Degron (RAF2181), intestine-specific AID DAF-2::degron (RV6), and pan-neuron-specific AID DAF-2::degron (RV8) were grown on OP50 until the L4 stage (approximately 18 × 90 mm NGM petri dishes with 500–800 animals feeding OP50 per plate). At the L4 stage, FUdR (50 μM) was top-coated on the plates. On day 15, animals from 6 petri dishes were collected as “pretreated group” (D15 animals). The rest of the animals were divided into two batches with 6 petri dishes each. One batch was treated with DMSO and another with 1 mM auxin on day 15. Animals were harvested 4 days after the treatment as “DMSO-treated group” and “auxin-treated group” (D19 animals). *C. elegans* were collected in M9 buffer, washed two times with the buffer, and then as a pellet flash-frozen for storage at − 80 °C until RNA isolation. RNA isolation was done using the Qiagen RNeasy Mini Kit (#74004) according to the manufacturer’s instructions. Three biological replicates were collected for the experiment.

### RNAseq analysis

RNA concentration and purity were measured using a Nanodrop spectrophotometer and validated by Agilent 2100 Bio-analyzer. cDNA libraries were generated using the Illumina TruSeq Stranded Total RNA sample preparation protocol. Libraries were pooled and sequenced on an Illumina NovaSeq 6000 at a targeted depth of 20 million paired-end 150 bp reads per sample. Reads were aligned to the *C. elegans* WBcel235 reference genome and annotated using the WBcel235.109 GTF using the align and featureCounts functions from the Rsubread R package (version 2.3.7) [82]. Differential expression analysis was performed using the edgeR (3.30.3) and limma (3.44.3) R packages [83, 84]. ENSEMBL Gene IDs were mapped to gene symbols using the mapIds function from the AnnotationDbi package (1.51.1). Filtering was performed to exclude genes that had fewer than 5 reads mapping in at least 5 samples, followed by normalization using the trimmed mean of *M*-values method via the calcNormFactors function in edgeR. Differential expression was determined using the limma voom pipeline to generate linear models with empirical Bayes moderation. *P*-value correction was performed using a Bejamini-Hochberg false detection rate correction. Gene set enrichment analysis was performed using the enrichGO function from the clusterProfiler package (3.16.0) [85]. Raw reads are deposited in the National Institutes of Health Sequence Read Archive (SRA) under BioProject accession: PRJNA1072966 and processed data are in Supplementary Table 2.

### *C. elegans* stiffness and roughness

For nematode stiffness and roughness measures DAF-2::degron (RAF2181) *C. elegans* were aged in cohorts and lifespans were measured in parallel until the last day of measurement (D24 for DMSO and D36 for auxin) when all remaining *C. elegans* were measured. On day 12, auxin (1 mM final concentration) or DMSO (control) was applied as a top coat to the plates. *C. elegans* stiffness was measured on days 18, 24 (DMSO and auxin), and days 30 and 36 (for auxin), and cuticle roughness at day 18 (DMSO and auxin) and day 30 (for auxin) using atomic force microscopy (NanoWizard III JPK Bruker) as described previously [35]. Briefly, for stiffness measures, *C. elegans* were paralyzed in a droplet of 15 mg/ml BDM, *C. elegans* for roughness measures were fixed in 4% PFA until assayed. Paralyzed or fixed *C. elegans* were transferred to to a 4% agarose pad in a 30 mm petri dish, and glued to the agarose at the head and tail using tissue glue (DERMABOND). Force measurements were taken from the glue-free neck and hip region of the *C. elegans* using a cantilever with a 10 µm diameter glass bead as indenter with a calibrated stiffness of *K* = 9.2–10.06 N/m (CP-µMasch NSC12/tipless/NoAl produced by sQUBE www.sQUBE.de). Topographical images to determine cuticle roughness were acquired from the same regions in imaging mode (265px, scanning speed 0.7 Hz, deflection at 0.2–0.4 V) using very soft cantilevers designed for imaging in contact mode (qp-CONT-10 k = 0.1 N/m; nanosensors). All measurements were taken in liquid (M9) and at room temperature. The cantilever stiffness and sensitivity were calibrated before each set of measurements using the JPK calibration software. The raw force-indentation data was analyzed using the JPK analysis software, by setting the baseline and contact point and subtracting the cantilever bending. The Young’s Moduli were estimated using the Hertz/Sneddon model for contact mechanics using the JPK software. All topographical images were flattened at 2° using the plane fitting tool within the JPK software to correct for sample tilt and natural curvature of the *C. elegans*. The roughness of the cuticle was determined using the histogram function of the software by averaging the roughness (RMS roughness Rq) of 10 independent 1.5 μm^2^ areas within the annuli region of a topographical image of at least 11 *C. elegans*. Two to four topographical images per *C. elegans* were quantified and averaged.

## Protein aggregation assays

### PAB-1, LBP-2, and RHO-1 aggregation

For aggregation assessment, DCD421, DCD423, and DCD434 were age-synchronized by transferring adults to 20 °C and selecting their progeny at the L4 stage. Experiments were done in the absence of FUdR by transferring to new plates every other day. Aggregation levels were determined using a Leica fluorescence microscope M165 FC with a Planapo 2.0 × objective. Aggregation was quantified following pre-set criteria. Animals overexpressing *Pmyo-2::tagRFP::PAB-1* were divided into three categories: animals with up to ten (low aggregation) or more than ten (medium aggregation) fluorescent-labeled PAB-1 puncta in the pharyngeal posterior bulb, or more than ten (high aggregation) fluorescent-labeled PAB-1 puncta in the pharyngeal anterior bulb, as previously described [56]. Animals overexpressing *Plbp-2::lbp-2::tagRFP* were divided into three categories: animals with no fluorescent-labeled LBP-2 puncta (low aggregation), up to ten puncta (medium aggregation) and more than ten puncta (high aggregation) in the head region as previously described [57]. Animals expressing *Pmyo2::rho-1::venus* were divided into four categories: animals with < 10 aggregates in the procorpus and anterior pharyngeal bulb (A), animals with > 10 aggregates in one side of the procorpus (B), animals with > 10 aggregates in both sides of the procorpus (C) and animals with > 10 aggregates in the anterior pharyngeal bulb (D). For confocal analysis using a Leica Stellaris 8 with the HC PL APO CS2 63x/1.30 NA glycerol objective, worms were mounted onto slides with 2% agarose pads using 2 µM levamisole for anesthesia and tagRFP-labeled proteins were detected using 555 nm as excitation and an emission range from 565 to 620 nm.

For statistics, a Chi-square test was performed in GraphPad Prism (8.0.1), comparing treated vs. control groups at each timepoint.

### PolyQ35 aggregation

To quantify PolyQ35 aggregates, ARM415 animals were cultured at 20 °C on NGM plates containing 50 μM FUdR until day 8/9 of adulthood (or when 25% of the animals had stopped moving), at which point they were treated with either DMSO or 1 mM auxin, and incubated for an additional two/three days at 20 °C. After two/three days, animals were anesthetized using tetramisole dissolved in M9 at a final concentration of 4 mM mounted on 2% agarose pads. Images were collected either on a Zeiss SteREO Lumar.V12 stereoscope (Thornwood, NY, USA) using a DAPI filter set (Trial 1), or an upright bright field fluorescence microscope (Tritech Research, model: BX-51-F) equipped with an ET485/10 × narrow band excitation filter (25 mm, ringed; Chroma Technology, catalog number: ET485/10x) [86]. Images were acquired using the “The Imaging Source” camera model: DFK 23UX236, with IC Capture 2.4 software. (Trial 2). Mean fluorescence intensity was quantified by manually tracing around each animal in ImageJ v1.53 k. The number of aggregates was determined using the “Analyze Particles” tool in ImageJ (threshold = 100, size = 10–400, Circ = 0.3–1).

## Heat stress and osmotic stress

RAF2181 DAF-2::degron *C. elegans* were kept at 20 °C before being assayed. For mid-life (day 12) heat stress, *C. elegans* were transferred to 37 °C for 3 h and transferred back to 20 °C afterward. Then, *C. elegans* were scored for survival 24 h later. For osmotic stress, *C. elegans* were transferred to plates with increased NaCl concentration (400 mM) and scored for survival 48 h later [42]. *C. elegans* were scored as dead if they were unresponsive to physical touch using a platinum wire. Late-life (day 24) assays were performed similarly; however, the heat stress was shorter at 2.5 h, and for osmotic stress, animals were scored after 24 h instead of 48 h.

## Oxidative stress

### Oxidative stress assay using arsenite with automated wormtracker

Resistance to oxidative stress was quantified using the wMicroTracker (NemaMetrix) platform. Briefly, animals were resuspended in M9 at indicated timepoints and washed three times with M9 by centrifugation. 30 – 40 C. elegans were pipetted into each well of a round-bottomed 96-well plate in either M9 or 14 mM sodium arsenite solution and assayed for 50 h. Each genotype was always measured both in M9 (ca. 2 wells) as well as arsenite (ca. 6 wells) in parallel to enable comparisons. Activity measurements were analyzed using GraphPad Prism (8.0.1). Shown is the average activity and the standard deviation of the wells. For statistics, the area under the curve for each group was calculated and then compared with each other using an ordinary one-way ANOVA and Tukey’s multiple comparisons test.

## Immunosenescence

Immune aging assays were performed as described previously [41] with minor modifications. Gravid adult *C. elegans* were allowed to lay eggs overnight to synchronize progeny on nematode growth media (NGM) plates seeded with E. coli OP50 bacteria. The progeny that reached L4 or the young pre-fertile adult stage were then transferred onto 0.25% DMSO-containing NGM plates treated with 50 µM 5-fluoro-2′-deoxyuridine (FUdR; Sigma-Aldrich). The animals were maintained on these plates at 20 °C for 4 days. For preparing day 8 adult *C. elegans*, day 4 adult *C. elegans* were transferred to NGM plates containing 0.25% DMSO or auxin (1 mM; indole-3-acetic acid; Alfa Aesar) and maintained for 4 more days. On day 4 or day 8, adult *C. elegans* were moved onto *Pseudomonas aeruginosa* (PA14)-seeded plates. PA14 standard slow-killing assays were performed as previously described [87]. Briefly, 5 µl of overnight PA14 liquid culture was seeded onto the center of 0.35% peptone NGM solid media. Plates were incubated at 37 °C for 24 h and subsequently at room temperature for over 8 h before use. *C. elegans* at different ages were simultaneously infected with PA14 on the plates containing 50 µM FUdR at 25 °C. After infection (time = 0 on survival curves), the deaths of animals were scored at least once a day by gently prodding with a platinum wire. Significance was tested using the Log-rank (Mantel-Cox) test. Statistics were done in GraphPad Prism (8.0.1).

### Supplementary information

Below is the link to the electronic supplementary material.

Supplementary file1 (PDF 16.9 MB)

Supplementary file2 (XLSX 1203 KB)

Supplementary file3 (PDF 129 KB)

Supplementary file4 (XLSX 7473 KB)

Supplementary file5 (XLSX 141 KB)
