# Supplementary material for: Improved resilience and proteostasis mediate longevity upon DAF-2 degradation in old age
Source: GeroScience. 2024 Jun 20;46(5):5015–36. doi: 10.1007/s11357-024-01232-x (PMC11335714; doi:10.1007/s11357-024-01232-x)
Supplement: Supplementary file 1 — Supplementary file1 (PDF 16.9 MB) [file 11357_2024_1232_MOESM1_ESM.pdf]

Supplementary Figures

Title: Improved resilience and proteostasis mediate longevity upon DAF-2 degradation in old age

Journal: GeroScience

Authors: Adrian Molière, Ji Young Cecilia Park, Anita Goyala, Elena M. Vayndorf, Bruce Zhang, Kuei Ching Hsiung, Yoonji Jung, Sujeong Kwon, Cyril Statzer, David Meyer, Richard Nguyen, Joseph Chadwick, Maximilian A. Thompson, Björn Schumacher, Seung-Jae V. Lee, Clara L. Essmann, Michael R. MacArthur, Matt Kaeberlein, Della David, David Gems, Collin Y. Ewald

Corresponding author: Colliln Y. Ewald, collin-ewald@ethz.ch

Supplementary Fig. 1: Bacterial growth has a limited effect on DAF-2 AID-mediated lifespan extension.

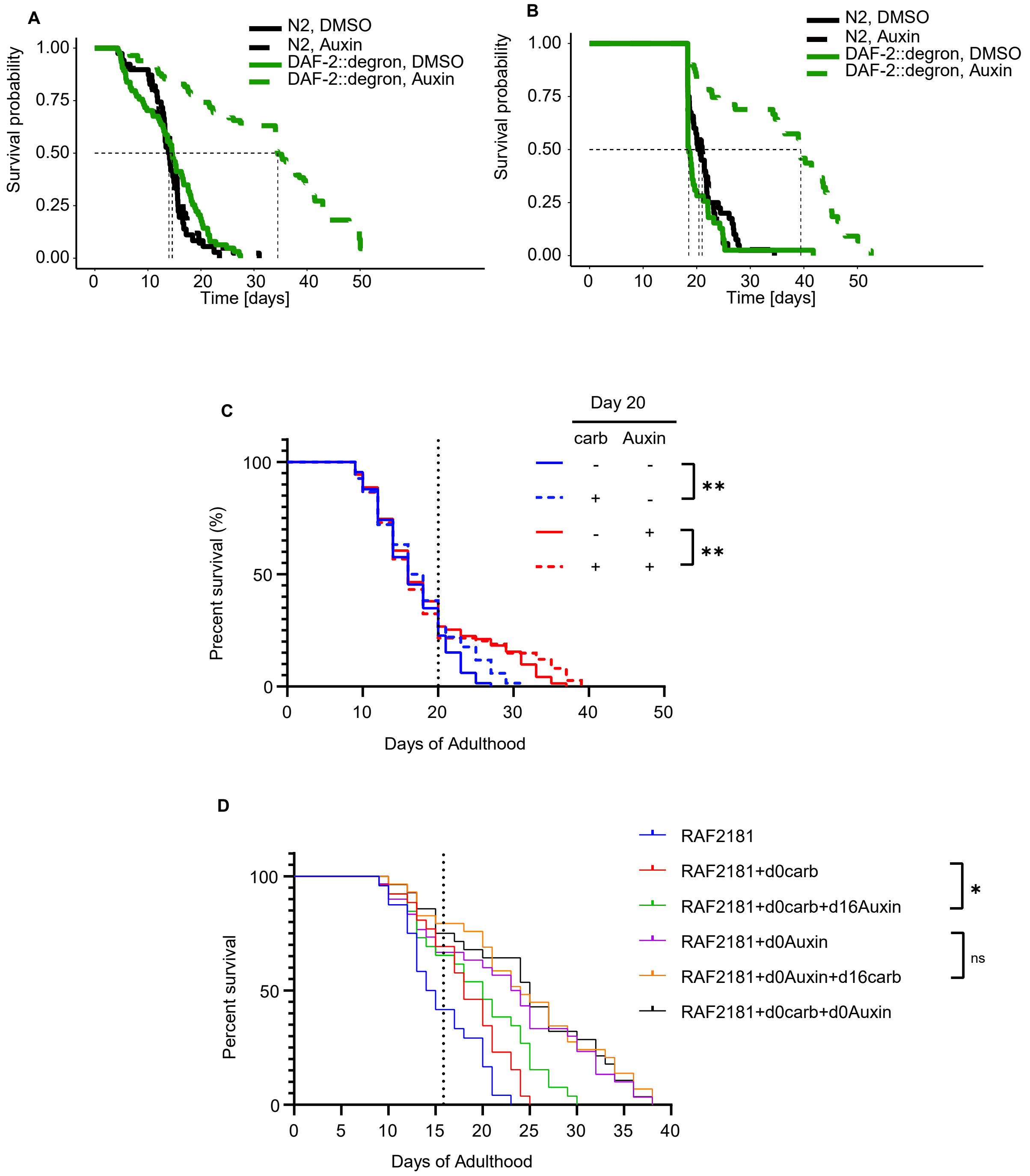

A Increased lifespan at 20°C after DAF-2 AID on day 5 in whole-body DAF-2::degron *C. elegans* compared to DMSO control. *C. elegans* were transferred to either DMSO or auxin (1 mM) - containing plates on day 5 of adulthood. Bacteria were heat-killed before plates were seeded. Survival was assessed in the automated lifespan machine.
B Increased lifespan at 20°C after DAF-2 AID on day 15 in whole-body DAF-2::degron *C. elegans* compared to DMSO control. *C. elegans* were transferred to either DMSO or auxin (1 mM) - containing plates on day 15 of adulthood. Bacteria were heat-killed before plates were seeded. Survival was assessed in the automated lifespan machine.
C Carbenicillin (500 mM) treatment on day 20 of adulthood (dotted line) increased lifespan both together with auxin (1 mM) (**: p =0.0091) and control DMSO treatment (**: p =0.0065) in whole-body DAF-2::degron *C. elegans* at 20°C. Determined using log-rank (Mantel-Cox) pairwise comparison.
D DAF-2 AID on day 16 of adulthood increased the lifespan of whole-body DAF-2::degron *C. elegans* at 20°C. The survival curves of *C. elegans* treated with carbenicillin (500 mM) and/or auxin (1 mM) at day zero or day 16 of adulthood are shown. The dotted line indicates the day 16 treatment time point. *: p = 0.0124; ns: p =0.9485. Determined using log-rank (Mantel-Cox) pairwise comparison. For statistics and additional information, see Supplementary Table 1.

Supplementary Fig. 2: No improvement in pathologies following DAF-2 AID on day 10 or day 14 of adulthood.

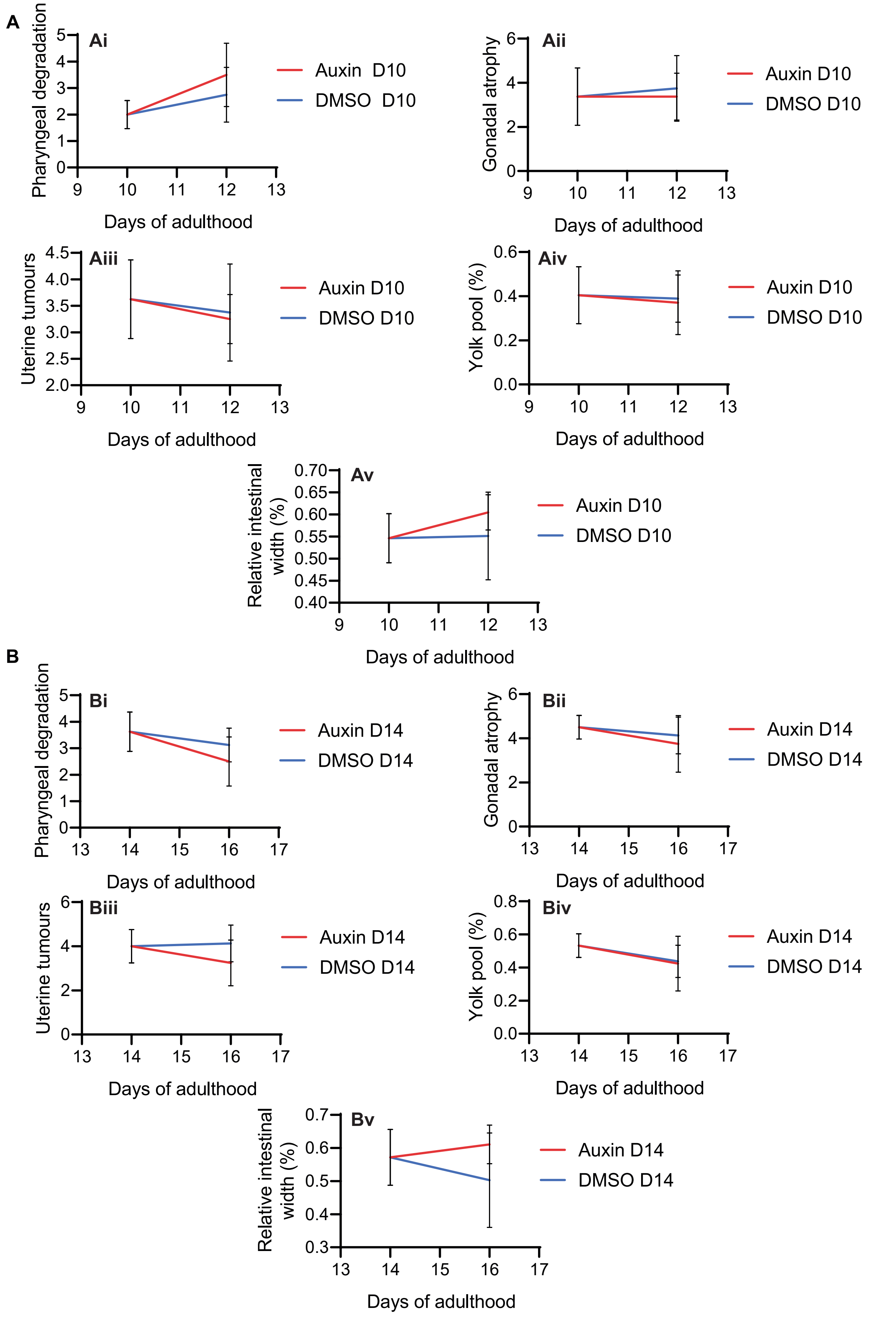

DAF-2 AID on day 10 (**A**) or day 14 (**B**) of adulthood does not affect senescent pathologies. Whole-body DAF-2::degron *C. elegans* were treated with auxin or DMSO to a final concentration of 1 mM on day 10 and day 14 and subsequently scored on the following days for pharyngeal degradation (**i**), and gonadal atrophy (**ii**), uterine tumors (**iii**) (scored on a scale from 1 to 5), as well as the relative size of yolk pools (**iv**) and intestinal width (**v**).

Supplementary Fig. 3: No improvement in pharyngeal pumping following DAF-2 AID on day 12 of adulthood.

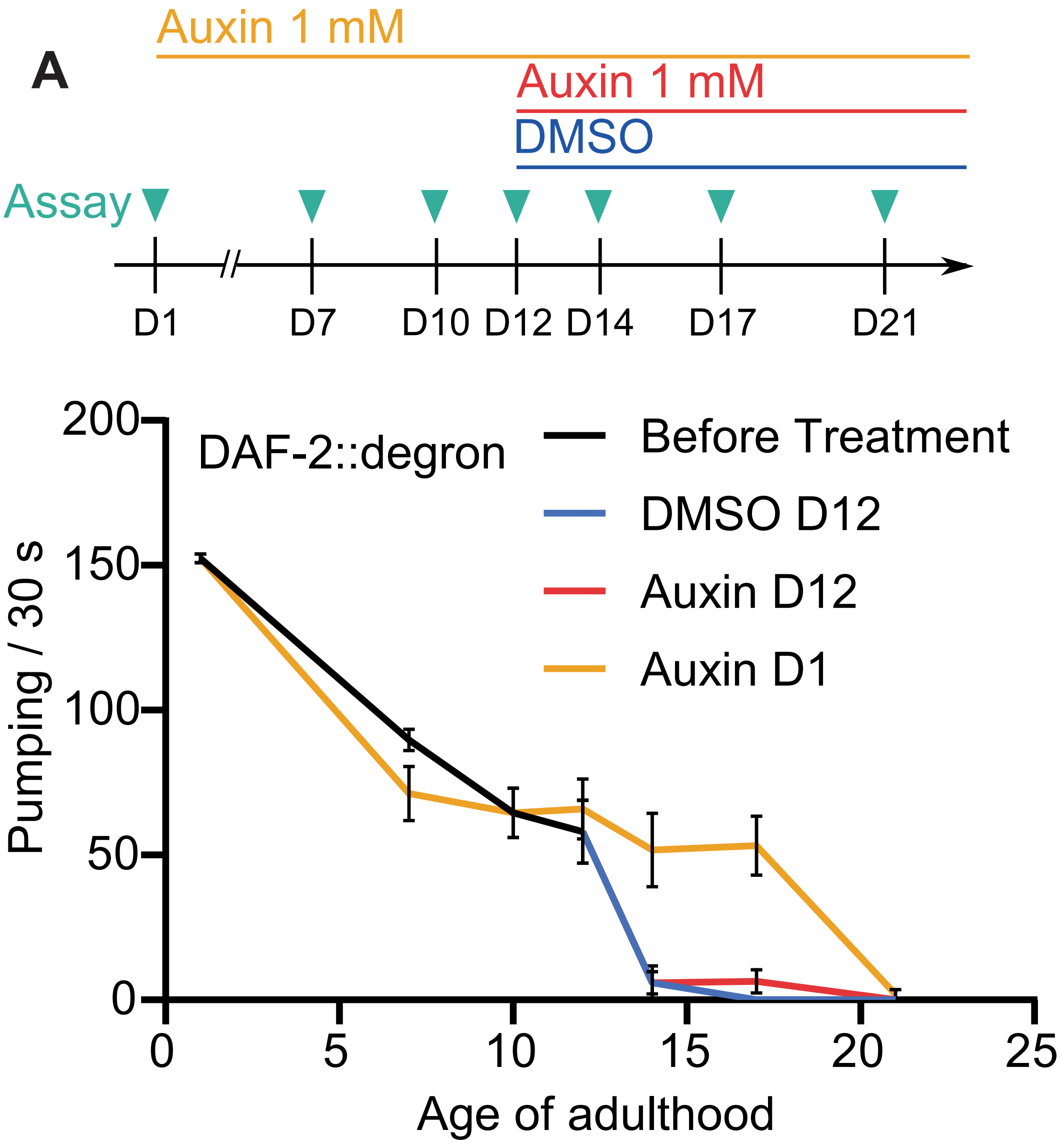

A No effect on the decline in pharyngeal pumping rate after DAF-2 AID on day 12 compared to DMSO control. Whole-body DAF-2::degron *C. elegans* were treated with auxin on day 1 or with either DMSO or auxin to a final concentration of 1 mM on day 12 and subsequently scored on days 14, 17, and 21. Error bars represent mean and SEM. Pooled result of 2 independent biological repeats. For individual repeats, see Source Data File 1.

Supplementary Fig. 4: Maintenance of cuticle integrity beyond day 30 by DAF-2-AID on day 12.

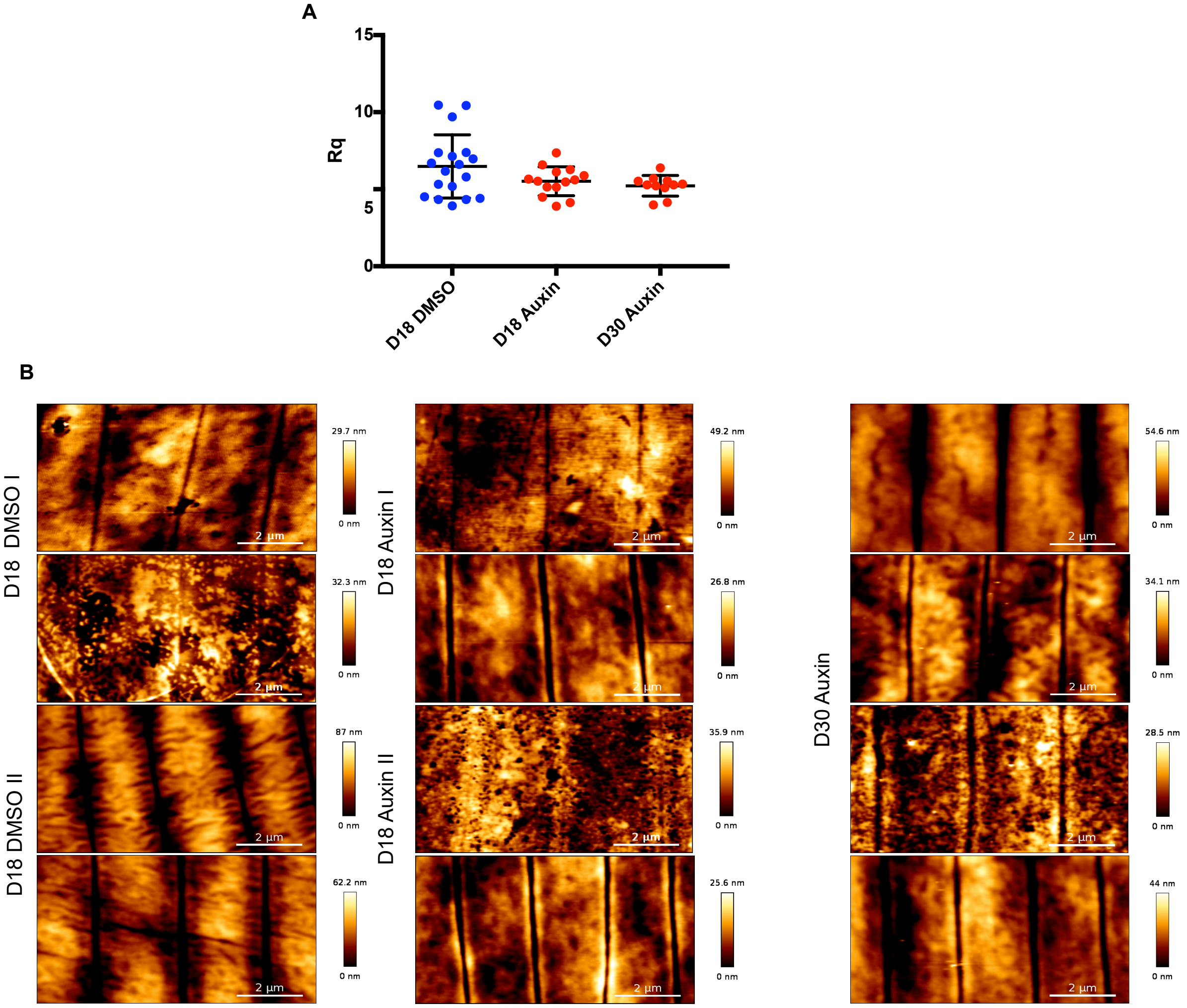

A Roughness quantification of topographical images presented as RMS roughness Rq ± standard deviation on day 18 (DMSO and auxin) or day 30 (auxin). Whole-body DAF-2::degron *C. elegans* were treated with either DMSO or auxin to a final concentration of 1 mM on day 12 and subsequently scored on days 18 or 30. (n = 18, 14, 11 for DMSO D18, auxin D18 or auxin D30 respectively)
B Representative AFM cuticle topography images used in A for roughness quantification. Images are from two independent experiments for day 18 (DMSO and auxin), and from one for day 30 (auxin).

Supplementary Fig. 5: Moderate overlap between changes seen upon DAF-2 AID on day 1 and day 15

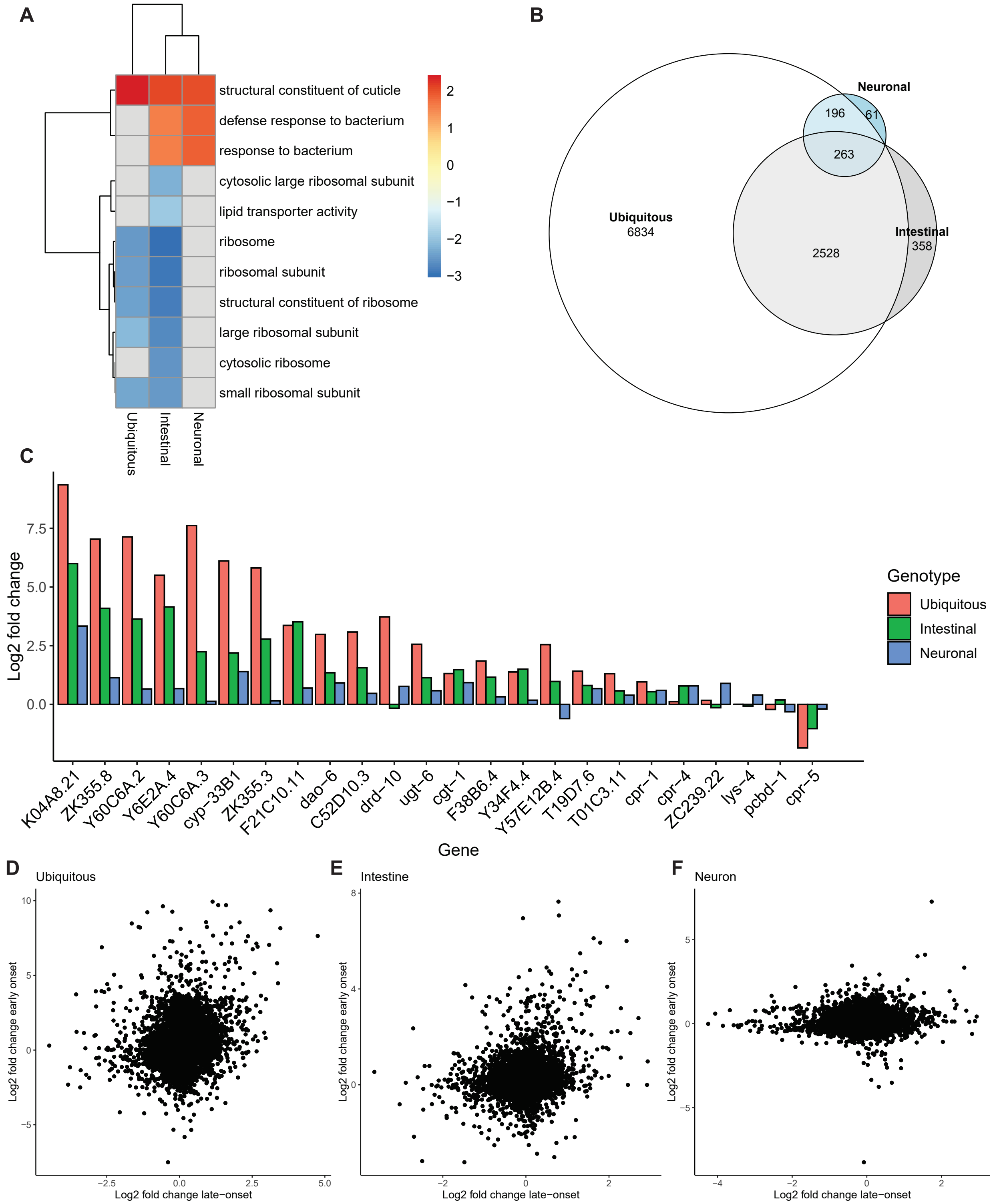

Figure shows the reanalysis of data from Zhang et. al. (23).
A Shown is the number of significant differentially expressed genes for each *C. elegans* strain and their overlap upon DAF-2 AID on day 1.
B Heatmap of differentially expressed genes grouped in GO terms, the scale represents fold-changes.
C Shown is the differential expression of genes upon DAF-2 AID on day 1 that previously showed the greatest differential expression on day 15 in the three *C. elegans* strains (whole-body DAF-2::degron, neuronal DAF-2::degron, intestinal DAF-2::degron).
D-F Shown is the similarity between changes in gene expression upon DAF-2 AID on day 1 (early onset) and day 15 (late onset) in the three *C. elegans* strains (whole-body DAF-2::degron (**D**), neuronal DAF-2::degron (**E**), intestinal DAF-2::degron (**F**)). Each dot represents a gene.

Supplementary Fig. 6: Late-life AID of DAF-2 also improved stress survival

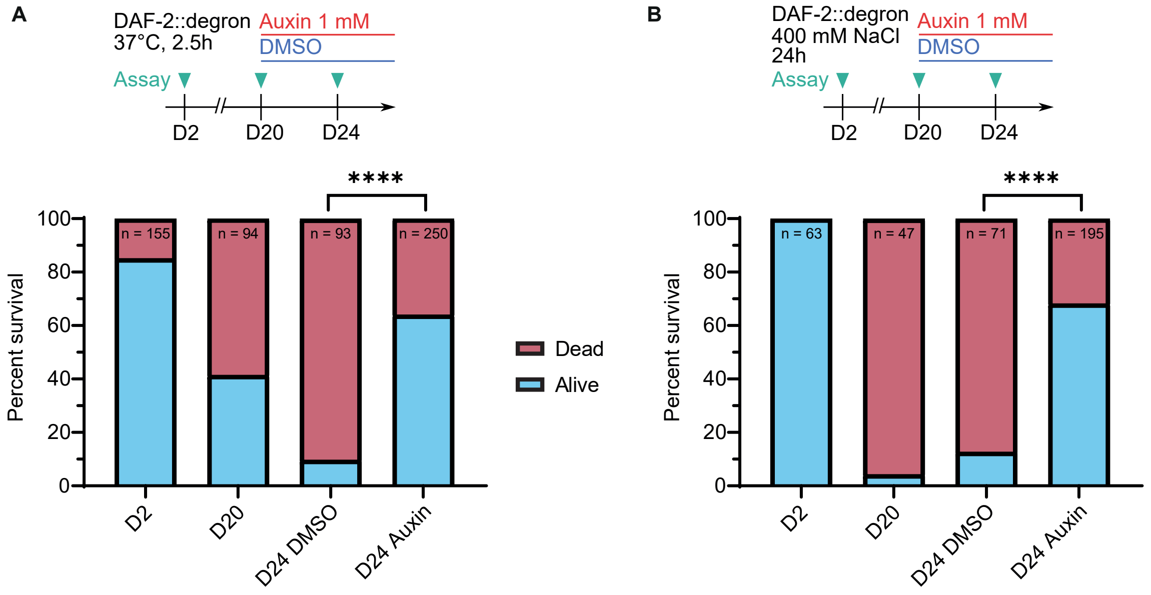

A DAF-2 AID on day 20 of adulthood reversed the age-related loss of heat stress resistance in *C. elegans* compared to DMSO-treated controls on day 24 of adulthood. Whole-body DAF-2::degron *C. elegans* were treated with either DMSO or 1 mM auxin on day 20 of adulthood. Four days later, on day 24, DAF-2::degron *C. elegans* were heat-shocked for 2.5h at 37°C and scored for survival 24h later. ****: $p < 0.0001$. Determined using Fisher's exact test. Three biological repeats, with two reaching statistical significance. Shown is the repeat with the highest total n. For additional trials and information, see Source Data File 1.
B DAF-2 AID on day 20 of adulthood reversed the age-related loss of osmotic stress resistance in *C. elegans* compared to DMSO-treated controls on day 24 of adulthood. Whole-body DAF-2::degron *C. elegans* were treated with either DMSO or 1 mM auxin on day 20 of adulthood. On day 24, DAF-2::degron *C. elegans* were transferred to fresh 400 mM NaCl plates and scored for survival 24h later. ****: $p < 0.0001$. Determined using Fisher's exact test. Three biological repeats, with two reaching statistical significance. Shown is the repeat with the highest total n. For additional trials and information, see Source Data File 1.

Supplementary Fig. 7: Trend towards less total aggregated protein load after DAF-2 AID

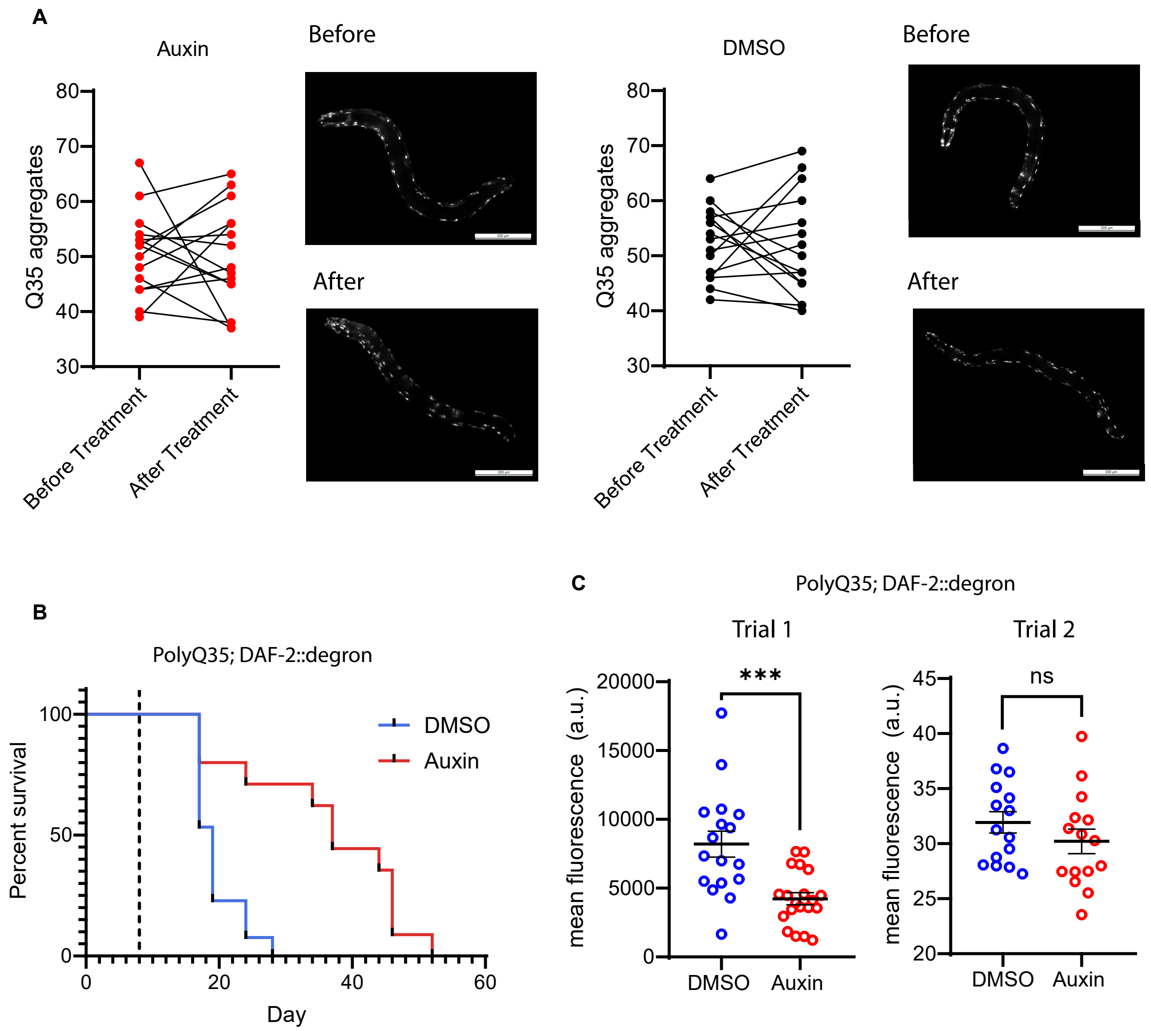

A DAF-2 AID on day 8 has no effect on the number of Q35 aggregates in poly-Q35 DAF-2::degron *C. elegans*. First imaging and transfer to either DMSO or 1 mM auxin-containing plates on day 8. On day 10, the same individuals were imaged again. Images show example individuals for both DMSO and auxin-treated *C. elegans*. Scale bar: 200 µm.
B DAF-2 AID at day 8 increases the lifespan of poly-Q35 DAF-2::degron *C. elegans*. Shown is the subsequent survival of the same *C. elegans* imaged in (A). Scale bar: 200 µm. For additional information see Supplementary Table 1.
C Trend towards reduced fluorescence intensity after DAF-2 AID in poly-Q35 DAF-2::degron *C. elegans*. Trial one: Transfer to auxin or DMSO-containing plates on D9, imaging on D12. Trial two: Transfer to auxin or DMSO-containing plates on D8, imaging on D10. Error bars represent mean and SEM. ***: $p < 0.001$.

Supplementary Fig. 8: Further RHO-1::venus aggregation halted and partially cleared with DAF-2 AID

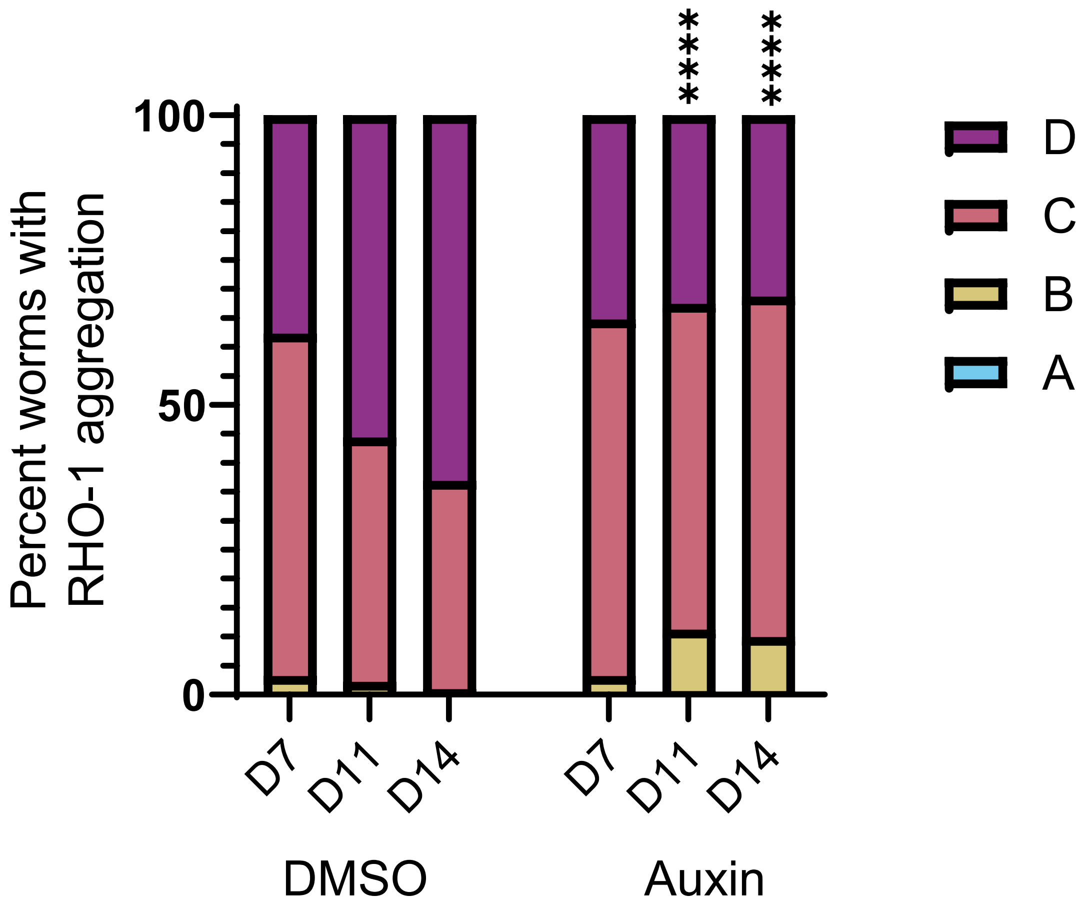

Whole-body DAF-2 AID on Day 7 resulted in a halt in RHO-1::venus aggregation and a small subset of animals showed clearance (animals transitioned from C to B). Animals were scored on Day 7 and then transferred to either DMSO or 1 mM auxin-containing plates the same day. The RHO-1 aggregation status was then subsequently scored on D11 and D14. Significance determined by Chi-squared test, ****: $p < 0.0001$.
