# Supplementary material for: Improved resilience and proteostasis mediate longevity upon DAF-2 degradation in old age
Source: GeroScience. 2024 Jun 20;46(5):5015–36. doi: 10.1007/s11357-024-01232-x (PMC11335714; doi:10.1007/s11357-024-01232-x)
Supplement: Supplementary file 3 — Supplementary file3 (PDF 129 KB) [file 11357_2024_1232_MOESM3_ESM.pdf]

Supplementary Discussion

Title: Improved resilience and proteostasis mediate longevity upon DAF-2 degradation in old age

Journal: GeroScience

Authors: Adrian Molière, Ji Young Cecilia Park, Anita Goyala, Elena M. Vayndorf, Bruce Zhang, Kuei Ching Hsiung, Yoonji Jung, Sujeong Kwon, Cyril Statzer, David Meyer, Richard Nguyen, Joseph Chadwick, Maximilian A. Thompson, Björn Schumacher, Seung-Jae V. Lee, Clara L. Essmann, Michael R. MacArthur, Matt Kaeberlein, Della David, David Gems, Collin Y. Ewald

Corresponding author: Colliln Y. Ewald, collin-ewald@ethz.ch

Table 1: Supplementary Table 1: Top differentially expressed genes upon late-life AID of DAF-2. Information from WormBase WS290 (1).

**Name**	**Function**	**Human ortholog**	**Category**
Y71F9AM.11	Uncharacterized		
ZK355.3	Uncharacterized, target of DAF-2	similarity to Interleukin-7 receptor	
Y71F9AM.8	Uncharacterized, target of DAF-2		
B0284.5 pseudogene	Uncharacterized, target of DAF-2		
spp-27	Pathogen defense		Pathogen defense
Y6E2A.4	Uncharacterized, target of DAF-2		
ZK355.8	Uncharacterized, target of DAF-2		
Y60C6A.3	Uncharacterized		
Y34F4.4	Uncharacterized		
F38B6.4	IMP biosynthesis pathway	GART	Metabolism
Y57E12B.4	Uncharacterized, target of DAF-2		
cpr-4	Radiation/ROS response, protein catabolism	cathepsin B	Radiation/ oxidative stress
T19D7.6	Uncharacterized, target of DAF-2		
T01C3.11	Uncharacterized		
dao-6	Oxidative stress response		Radiation/ oxidative stress
Y60C6A.2	Implicated in UV response		Radiation/ oxidative stress
F21C10.11	Uncharacterized, target of DAF-2		
cgt-1	Glucosylceramide biosynthesis	UGCG	Metabolism
cyp-33B1	Oxidoreductase, steroid hydroxylase, organic acid, and xenobiotic metabolism	CYP2B6, CYP2C8, CYP2J2	Metabolism
C52D10.3	Uncharacterized, target of DAF-2		
Y41D4B.15	Uncharacterized, target of DAF-2		
drd-10	Target of DAF-2, implicated in dietary restriction		Proteostasis
ugt-6	Glucuronosyltransferase activity	UGT3A1	Metabolism
pcbd-1	Predicted to enable 4-alpha-hydroxytetrahydrobiopterin dehydratase activity.	PCBD1	Metabolism
F08H9.4	Unfolded protein/ heat-shock response (2)		Proteostasis
cpr-5	Proteolysis and protein catabolic processes	cathepsin B	Proteostasis
lys-4	Pathogen response		Pathogen defense
ZC239.22	Uncharacterized, target of DAF-2		
cpr-1	Proteolysis and protein catabolic processes	cathepsin B	Proteostasis

1 RNAseq discussion

Of the 29 genes showing the greatest fold change in gene expression among old age neuronal, intestinal, and ubiquitous DAF-2 AID (Figure 3D), 15 were largely uncharacterized. The characterized genes fall into roughly 4 categories: pathogen defense, oxidative and radiation stress response, proteostasis, and metabolism. Two genes (lys-4 and spp-27) are predicted to play a role in the antimicrobial response and immunity (3). Three of the genes (cpr-4, dao-6, and Y60C6A.2) are predicted to play a role in oxidative or radiation stress response (4,5). The cathepsin B, cpr-4, which is downregulated upon AID of DAF-2, is of particular interest, as it is a radiation-induced bystander effect factor, exerting effects on other parts of the animal not exposed to radiation, causing genomic instability, stress responses, and altered apoptosis or cell proliferation (6). Three genes (cpr-1, cpr-5, and F08H9.4) can furthermore be loosely grouped together as being involved in proteostasis and the heat-shock response (2).
Finally, there are several genes that regulate metabolism, such as IMP-biosynthesis (F38B6.4), glucosylceramide biosynthesis (cgt-1), oxidoreductase activity and organic acid metabolism (cyb-33B1), glucuronosyltransferase activity (ugt-6), and 4-alpha-hydroxytetrahydrobiopepterin dehydratase activity (pcbd-1). One caveat of this analysis is that whole-body RNAseq was performed using strains with tissue-specific degradation of DAF-2. Thus, the effect on these specific tissues is likely diluted in the overall signal and the resulting signal probably reflects organismal effects of tissue-specific AID.

2 BiT age clock

Biological age prediction with BiT-age was carried out as described in Meyer and Schumacher, 2021 (`https://github.com/Meyer-DH/AgingClock`).
BiT-age predicts the survivor-bias-corrected biological age of the samples. The uncorrected chronological age in hours of a day 15 sample is 360 h. The uncorrected biological age would be 360 h as well, if the sequenced sample has a Median lifespan of 372 h (defined in (7) to be the Median lifespan of a WT worm population under standard conditions). A sample of a population of worms with a Median lifespan of 21 days (504 h) is 1.36x longer lived. After temporal rescaling with this correction factor of 1.36 the day 15 sample has a biological age of 360 h / 1.36 = 266 h.
In Meyer and Schumacher, 2021 an additional correction for a survivor bias was introduced, which decreases the predicted age of the sample. Briefly, a population of age-synchronized, isogenic worms contains biologically younger, respectively older, individuals. The biologically oldest worms will on average die earlier, leading to a survivor bias of an average biologically younger population of worms.

3 Stochastic data-based transcriptomic clock

The stochastic data-based clock predictions were carried out as described in (8) (`https://github.com/Meyer-DH/StochasticAgingClock`).
Briefly, the stochastic data-based clock is trained to predict how often normal distributed stochastic variation is added to a biologically young adult *C. elegans* RNA-seq sample.

Stochastic data-based clock results.

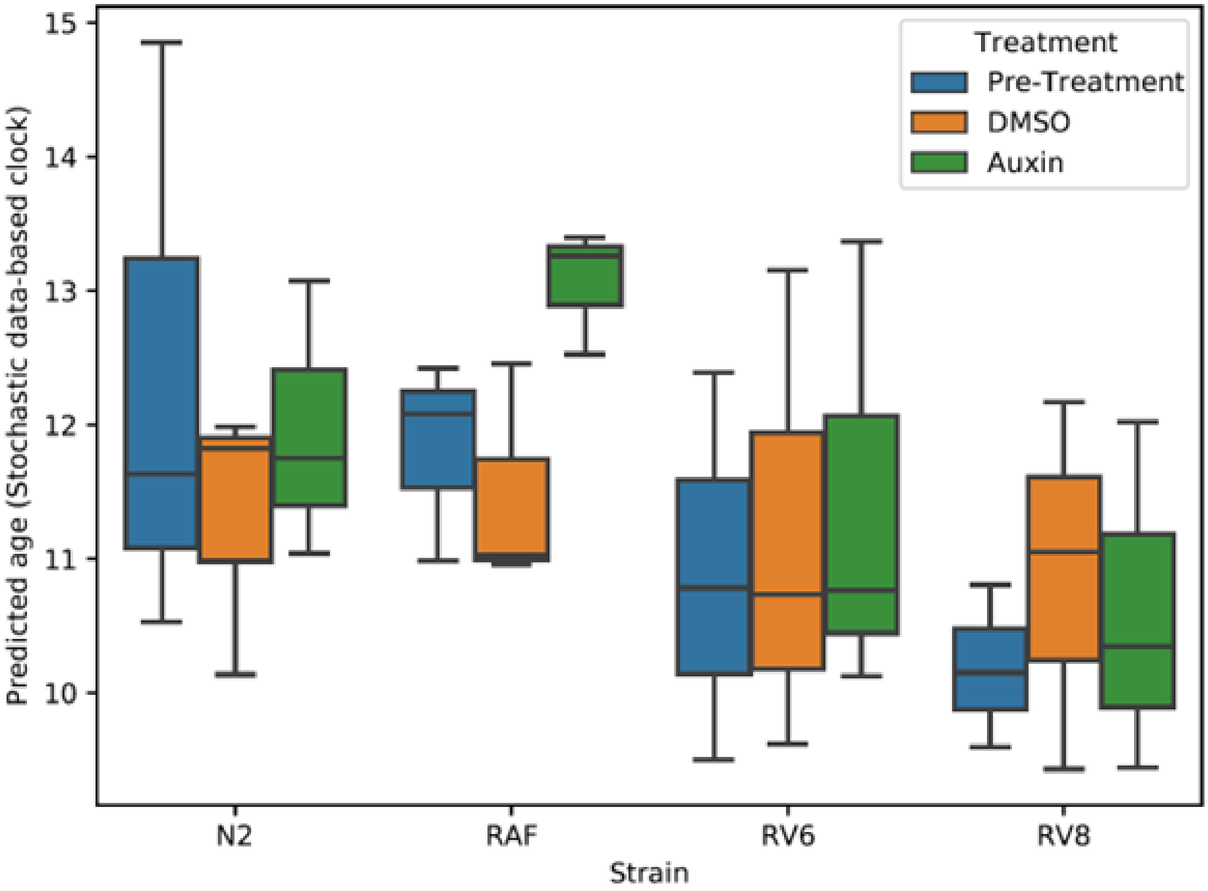

2-way ANOVA of stochastic data-based clock results.

Source	SS	DF	MS	F	p-unc	np2
Strain	13.563091	3	4.521030	2.592.562	0.076100	0.244753
Treatment	2.038324	2	1.019162	0.584433	0.565159	0.046441
Strain*Treatment	4.841185	6	0.806864	0.462692	0.828853	0.103680
Residual	41.852322	24	1.743847	NaN	NaN	NaN

4 References

1. Davis P, Zarowiecki M, Arnaboldi V, Becerra A, Cain S, Chan J, et al. WormBase in 2022—data, processes, and tools for analyzing Caenorhabditis elegans. Genetics. 2022;220(4):iyac003.
2. Shim J, Im SH, Lee J. Tissue-specific expression, heat inducibility, and biological roles of two hsp16 genes in Caenorhabditis elegans. FEBS Lett. 2003;537(1–3):139–45.
3. Roeder T, Stanisak M, Gelhaus C, Bruchhaus I, Grötzinger J, Leippe M. Caenopores are antimicrobial peptides in the nematode Caenorhabditis elegans instrumental in nutrition and immunity. Dev Comp Immunol. 2010;34(2):203–9.
4. Li A, Wei G, Wang Y, Zhou Y, Zhang X en, Bi L, et al. Identification of Intermediate-Size Non-Coding RNAs Involved in the UV-Induced DNA Damage Response in C. elegans. PLoS ONE. 2012;7(11):e48066.
5. Shin H, Lee H, Fejes AP, Baillie DL, Koo HS, Jones SJ. Gene expression profiling of oxidative stress response of C. elegans aging defective AMPK mutants using massively parallel transcriptome sequencing. BMC Res Notes. 2011;4(1):34.
6. Peng Y, Zhang M, Zheng L, Liang Q, Li H, Chen JT, et al. Cysteine protease cathepsin B mediates radiation-induced bystander effects. Nature. 2017;547(7664):458–62.
7. Meyer DH, Schumacher B. BiT age: A transcriptome-based aging clock near the theoretical limit of accuracy. Aging Cell. 2021;20(3):e13320.
8. Schumacher B, Meyer D. Accurate aging clocks based on accumulating stochastic variation. 2023;
